# Supplementary figures and images for: Mapping trends in insecticide resistance phenotypes in African malaria vectors
Source: PLoS Biol. 2020 Jun 25;18(6):e3000633. doi: 10.1371/journal.pbio.3000633 (PMC7316233; doi:10.1371/journal.pbio.3000633)

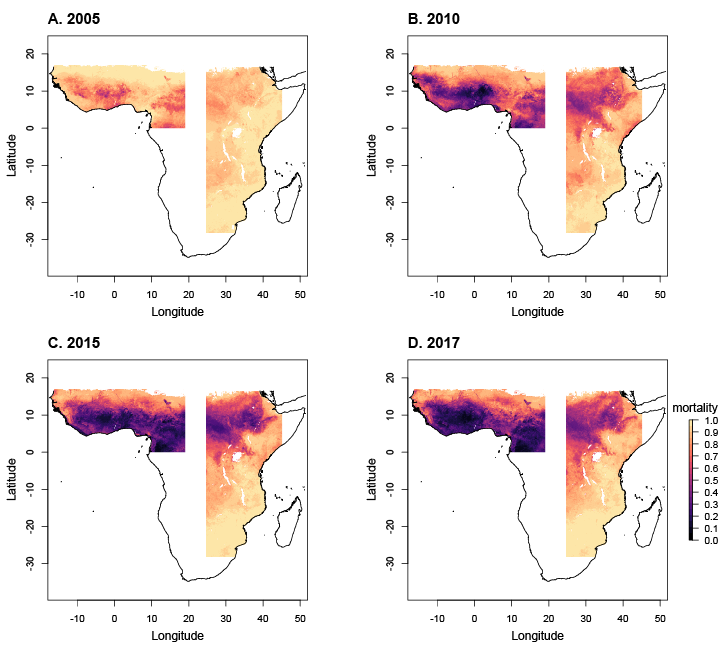

Supplement: S1 Fig — (A) 2005, (B) 2010, (C) 2015, and (D) 2017. See 10.6084/m9.figshare.9912623. (TIF) [file pbio.3000633.s001.tif]

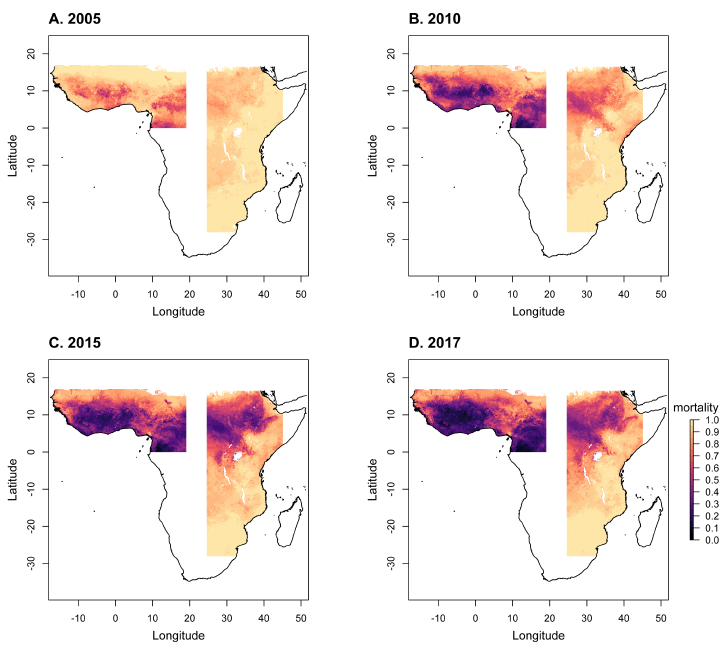

Supplement: S2 Fig — (A) 2005, (B) 2010, (C) 2015, and (D) 2017. See 10.6084/m9.figshare.9912623. (TIF) [file pbio.3000633.s002.tif]

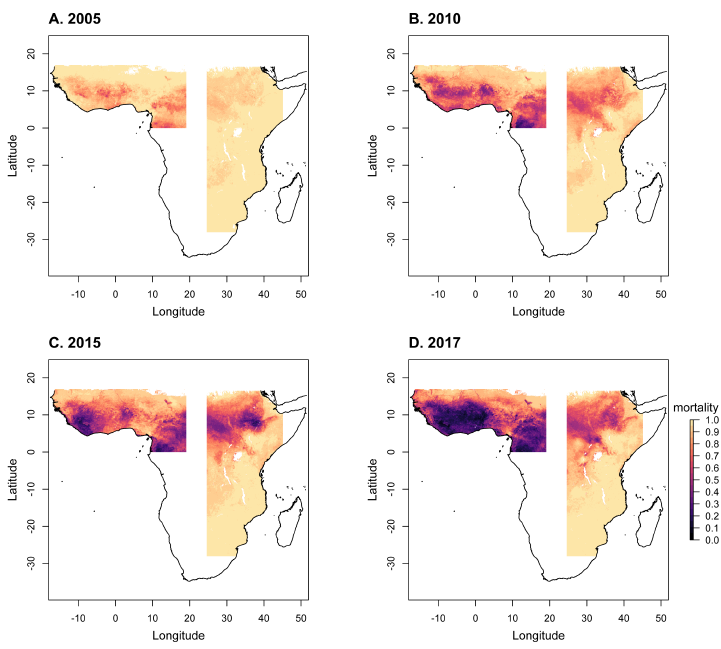

Supplement: S3 Fig — (A) 2005, (B) 2010, (C) 2015, and (D) 2017. See 10.6084/m9.figshare.9912623. (TIF) [file pbio.3000633.s003.tif]

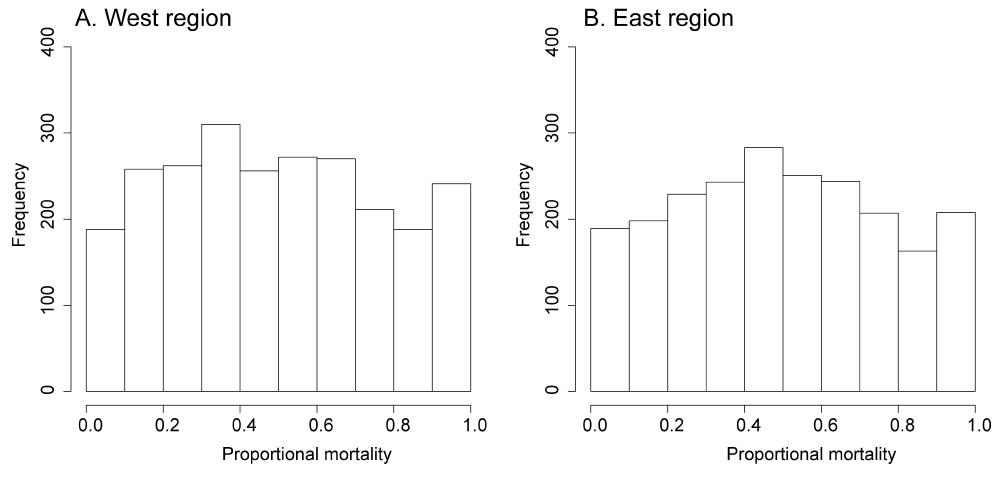

Supplement: S4 Fig — Numerical values are provided in S5 Data (10.6084/m9.figshare.9912623). (TIF) [file pbio.3000633.s004.tif]

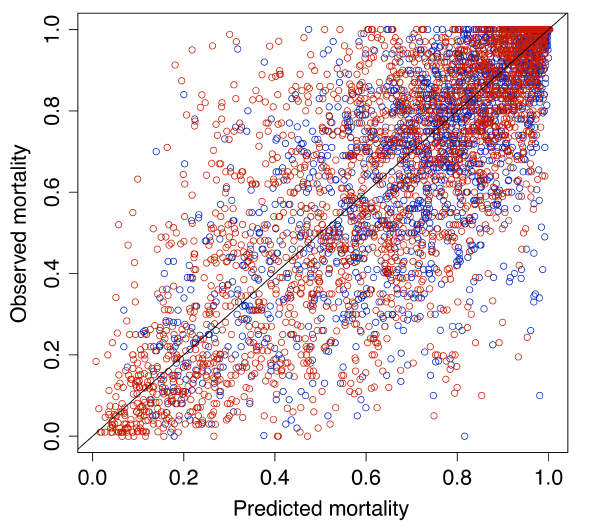

Supplement: S5 Fig — The vertical axis shows the corresponding value observed from the bioassay. Values for all data points for all pyrethroid types (deltamethrin, permethrin, λ-cyhalothrin, and α-cypermethrin) for the west region (red markers) and the east region (blue markers) are shown. The RMSE across all data values is 0.179 (RMSE = 0.191 for the data within the west region and RMSE = 0.166 for the data within the east region). Numerical values are provided in S6 Data (10.6084/m9.figshare.9912623). RMSE, root mean square error. (TIF) [file pbio.3000633.s005.tif]

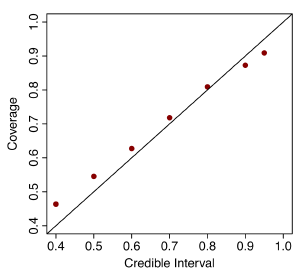

Supplement: S6 Fig — Numerical values are provided in S7 Data (10.6084/m9.figshare.9912623). CI, credible interval. (TIF) [file pbio.3000633.s006.tif]

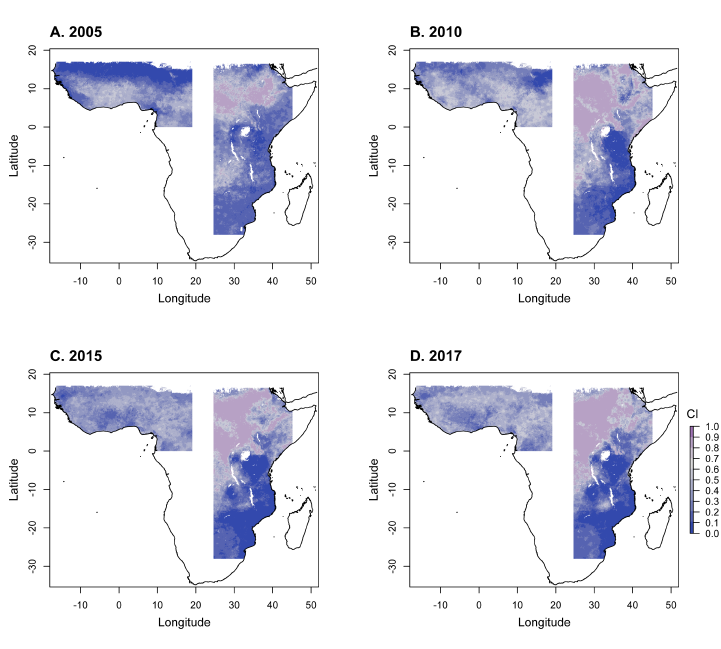

Supplement: S7 Fig — See 10.6084/m9.figshare.9912623. CI, credible interval. (TIF) [file pbio.3000633.s007.tif]

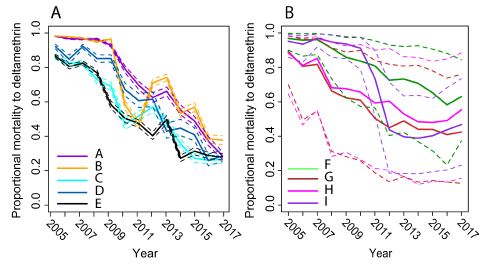

Supplement: S8 Fig — The predicted mean proportional mortality to deltamethrin over time for the point locations in the east (A) and west (B) regions that experienced the greatest overall increase in resistance from 2005 to 2017 (Fig 2; locations A, B, C, D, E, F, G, H, and I). Dashed lines show the 95% CIs of the predicted mean mortality. Numerical values are provided in S8 Data (10.6084/m9.figshare.9912623). CI, credible interval. (TIF) [file pbio.3000633.s008.tif]

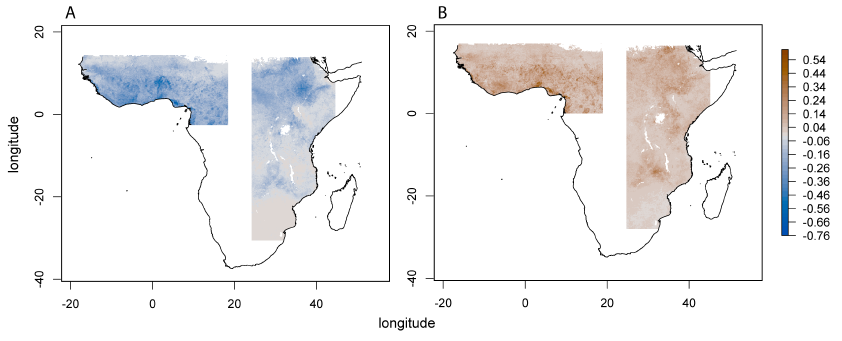

Supplement: S9 Fig — The maximum interannual change in the predicted mean mortality to deltamethrin over the time period 2005–2017 at each location within the west and east regions: (A) the maximum interannual decrease, (B) the maximum interannual increase. Interannual increases and decreases in predicted mortality are calculated as the difference in predictions between 2 consecutive years, for all years 2005 to 2017. See 10.6084/m9.figshare.9912623. (TIF) [file pbio.3000633.s009.tif]

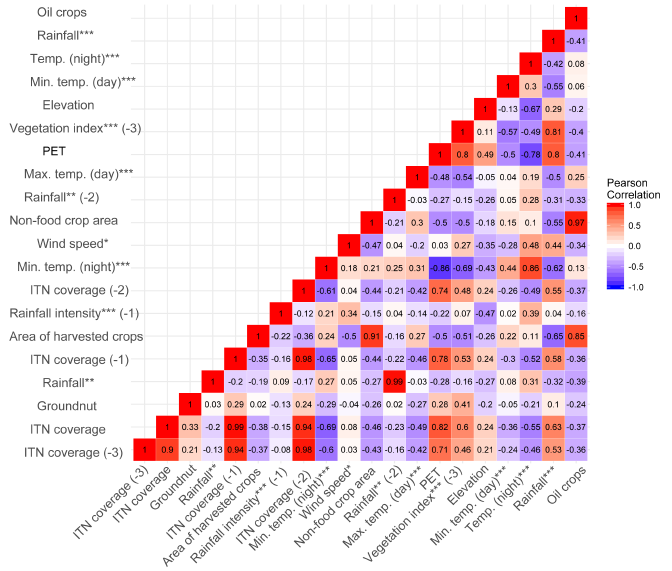

Supplement: S10 Fig — (TIF) [file pbio.3000633.s010.tif]

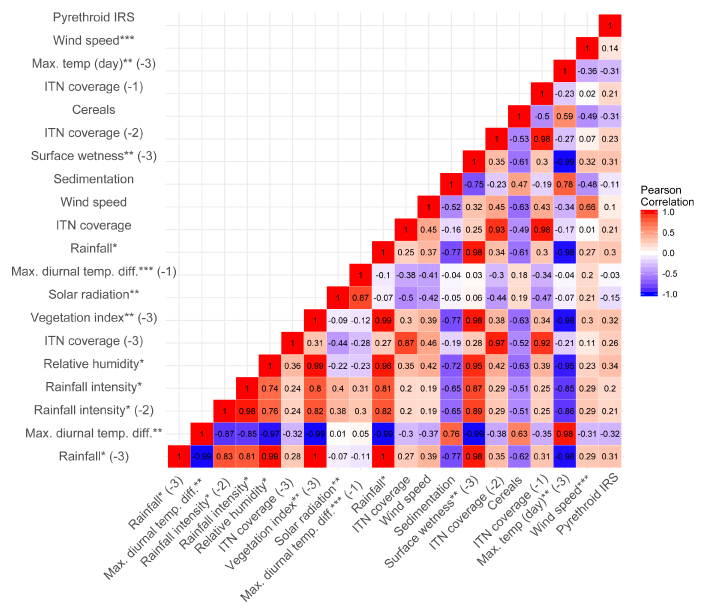

Supplement: S11 Fig — (TIF) [file pbio.3000633.s011.tif]
