# Supplementary material for: Mapping trends in insecticide resistance phenotypes in African malaria vectors
Source: PLoS Biol. 2020 Jun 25;18(6):e3000633. doi: 10.1371/journal.pbio.3000633 (PMC7316233; doi:10.1371/journal.pbio.3000633)
Supplement: S1 Table — Numbers in brackets are the 95% CIs. CI, credible interval. (DOCX) [file pbio.3000633.s012.docx]

| **Model for each insecticide type and region** | **Range^*^** | **Gaussian process variance $\sigma_{\omega}^{2}$** | **Measurement error variance $\sigma^{2}$** | **Temporal autocorrelation  ** |
| --- | --- | --- | --- | --- |
| **West region:** | | | | |
| Deltamethrin | 0.012 (0.0003,0.0036) | 0.42 (0.012,4.2) | 1.68 (1.57,1.84) | 0.02  (-0.6,0.65) |
| Permethrin | 0.083  (0.03,0.23) | 0.70  (0.34,1.26) | 1.21 (1.05,1.44) | -0.01  (-0.4,0.4) |
| λ-cyhalothrin | 0.007  (0.002, 0.22) | 2.1 (0.67,7.0) | 1.24  (1.1,1.42) | 0.13  (-0.39,0.6) |
| α-cypermethrin | 0.16  (0.06,0.34) | 1.93 (0.86,4.0) | 1.56  (1.2,2.0) | -0.11  (-0.62,0.41) |
| DDT | 0.06  (0.027,0.13) | 0.9 (0.4,1.85) | 1.23  (1.1,1.43) | -0.19  (-0.65,0.41) |
| **East region:** | | | | |
| Deltamethrin | 0.066  (0.042,0.1) | 1.31 (0.76,2.1) | 1.13  (1.0,1.25) | 0.12  (-0.28,0.52) |
| Permethrin | 0.004  (0.0011, 0.17) | 2.3 (0.19,20.4) | 1.35 (1.17,1.56) | -0.03  (-0.65,0.57) |
| λ-cyhalothrin | 0.09  (0.036,0.24) | 0.94 (0.38,2.66) | 1.51  (1.2,1.83) | -0.38  (-0.95,0.3) |
| α-cypermethrin | 0.028  (0.014, 0.052) | 4.45 (2.18,9.56) | 0.57  (0.35,1.0) | -0.3  (-0.83,0.44) |
| DDT | 0.063  (0.037, 0.1) | 1.63 (1.1,2.37) | 1.25  (1.0,1.5) | 0.57  (0.26,0.79) |

*Range is in the units of the unit sphere. An estimate in kilometres can by obtained by multiplying by the radius of the earth (~6371km).
