# Supplementary material for: Mapping trends in insecticide resistance phenotypes in African malaria vectors
Source: PLoS Biol. 2020 Jun 25;18(6):e3000633. doi: 10.1371/journal.pbio.3000633 (PMC7316233; doi:10.1371/journal.pbio.3000633)
Supplement: S2 Table — The unit of the transformed RMSE values corresponds to the (empirical logit and IHS-transformed) observations to which the models were fitted. IHS, inverse hyperbolic sine; RMSE, root mean square error. (DOCX) [file pbio.3000633.s013.docx]

| **Model** | **West Region** | | | **East Region** | | | **Combined** | | |
| --- | --- | --- | --- | --- | --- | --- | --- | --- | --- |
|  | **RMSE transformed** | **RMSE untransformed** | **MAE untransformed** | **RMSE transformed** | **RMSE untransformed** | **MAE untransformed** | **RMSE transformed** | **RMSE untransformed** | **MAE untransformed** |
| Gaussian process meta-model | 1.255 | 0.190 | 0.139 | 1.207 | 0.165 | 0.134 | 1.232 | 0.179 | 0.127 |
| XGB | 1.305 | 0.191 | 0.143 | 1.242 | 0.168 | 0.12 | 1.276 | 0.181 | 0.130 |
| RF | 1.36 | 0.197 | 0.150 | 1.278 | 0.175 | 0.115 | 1.32 | 0.191 | 0.136 |
| BGAM | 1.46 | 0.218 | 0.162 | 1.4 | 0.194 | 0.113 | 1.434 | 0.207 | 0.149 |
