# Supplementary material for: Mapping trends in insecticide resistance phenotypes in African malaria vectors
Source: PLoS Biol. 2020 Jun 25;18(6):e3000633. doi: 10.1371/journal.pbio.3000633 (PMC7316233; doi:10.1371/journal.pbio.3000633)
Supplement: S3 Table — The unit of the transformed RMSE values corresponds to the (empirical logit and IHS-transformed) observations to which the models were fitted. IHS, inverse hyperbolic sine; RMSE, root mean square error. (DOCX) [file pbio.3000633.s014.docx]

| **Model** | **West Region** | | | **East Region** | | | **Combined** | | |
| --- | --- | --- | --- | --- | --- | --- | --- | --- | --- |
|  | **RMSE transformed** | **RMSE untransformed** | **MAE untransformed** | **RMSE transformed** | **RMSE untransformed** | **MAE untransformed** | **RMSE transformed** | **RMSE untransformed** | **MAE untransformed** |
| Gaussian process meta-model | 1.19 | 0.168 | 0.122 | 1.3 | 0.166 | 0.097 | 1.24 | 0.167 | 0.111 |
| XGB | 1.28 | 0.170 | 0.129 | 1.46 | 0.171 | 0.104 | 1.35 | 0.170 | 0.117 |
| RF | 1.37 | 0.185 | 0.140 | 1.6 | 0.194 | 0.122 | 1.47 | 0.189 | 0.132 |
| BGAM | 1.56 | 0.226 | 0.175 | 1.8 | 0.219 | 0.14 | 1.67 | 0.222 | 0.160 |
