# Supplementary material for: Mapping trends in insecticide resistance phenotypes in African malaria vectors
Source: PLoS Biol. 2020 Jun 25;18(6):e3000633. doi: 10.1371/journal.pbio.3000633 (PMC7316233; doi:10.1371/journal.pbio.3000633)
Supplement: S4 Table — (DOCX) [file pbio.3000633.s015.docx]

| Constituent model | Meta-model for pyrethroid resistance | | Meta-model for DDT resistance | |
| --- | --- | --- | --- | --- |
|  | West region | East region | West region | East region |
| XGB | 0.54 | 0.44 | 0.5 | 0.4 |
| RF | 0.44 | 0.38 | 0.42 | 0.37 |
| BGAM | 0.22 | 0.19 | 0.37 | 0.31 |
