# Supplementary material for: Mapping trends in insecticide resistance phenotypes in African malaria vectors
Source: PLoS Biol. 2020 Jun 25;18(6):e3000633. doi: 10.1371/journal.pbio.3000633 (PMC7316233; doi:10.1371/journal.pbio.3000633)
Supplement: S5 Table — The 30 variables that were most highly ranked by XGB are shown. Definitions of each predictor variable are given in S9 Table. Variable name suffixes (-1), (-2) and (-3) denote time lags of 1, 2, and 3 years, respectively. One, two, and three asterisks denote the first, second, and third principal component, respectively, for variables available on a monthly time step. XGB, extreme gradient boosting model. (DOCX) [file pbio.3000633.s016.docx]

| **Predictor variable** | **Variable importance** | | | | | |
| --- | --- | --- | --- | --- | --- | --- |
|  | **XGB** | **Rank** | **RF** | **Rank** | **BGAM** | **Rank** |
| ITN coverage (-3) | 0.03627 | 1 | 0.00956 | 1 | 0.06730 | 2 |
| Rainfall** | 0.01536 | 2 | 0.00489 | 8 | 0.00267 | 96 |
| ITN coverage | 0.01184 | 3 | 0.00580 | 3 | 0.10952 | 1 |
| ITN coverage (-1) | 0.01042 | 4 | 0.00636 | 2 | 0.01061 | 14 |
| Rainfall** (-2) | 0.01004 | 5 | 0.00499 | 7 | 0.00000 | 336 |
| Rainfall intensity*** (-1) | 0.00987 | 6 | 0.00555 | 4 | 0.00462 | 63 |
| Elevation | 0.00947 | 7 | 0.00371 | 51 | 0.00207 | 119 |
| ITN coverage (-2) | 0.00945 | 8 | 0.00544 | 5 | 0.00543 | 46 |
| Vegetation index*** (-3) | 0.00917 | 9 | 0.00448 | 14 | 0.00157 | 146 |
| Area of harvested crops | 0.00900 | 10 | 0.00428 | 16 | 0.01735 | 6 |
| Pulse crops | 0.00832 | 11 | 0.00402 | 27 | 0.00027 | 277 |
| Solar radiation*** | 0.00804 | 12 | 0.00387 | 34 | 0.00129 | 160 |
| Area of non-food crops | 0.00779 | 13 | 0.00422 | 20 | 0.00732 | 30 |
| Groundnut crops | 0.00772 | 14 | 0.00373 | 44 | 0.02329 | 4 |
| Diurnal temperature difference* (-1) | 0.00724 | 15 | 0.00397 | 30 | 0.00192 | 128 |
| Palm oil crops | 0.00670 | 16 | 0.00316 | 93 | 0.00262 | 97 |
| Rainfall*** | 0.00667 | 17 | 0.00372 | 49 | 0.00497 | 54 |
| Temperate fruit crops | 0.00664 | 18 | 0.00485 | 10 | 0.00019 | 293 |
| Maximum night time temperature*** (-2) | 0.00654 | 19 | 0.00485 | 9 | 0.00017 | 295 |
| Minimum day time temperature*** (-3) | 0.00650 | 20 | 0.00423 | 19 | 0.00533 | 48 |
| Oil crops | 0.00621 | 21 | 0.00375 | 39 | 0.00599 | 42 |
| Rainfall erosivity | 0.00604 | 22 | 0.00410 | 24 | 0.00274 | 92 |
| Relative humidity*** | 0.00600 | 23 | 0.00415 | 23 | 0.00056 | 232 |
| Rainfall intensity* (-2) | 0.00599 | 24 | 0.00372 | 48 | 0.00289 | 85 |
| Surface wetness*** | 0.00595 | 25 | 0.00374 | 42 | 0.00278 | 90 |
| Maximum night time temperature*** (-3) | 0.00590 | 26 | 0.00500 | 6 | 0.00222 | 113 |
| Vegetation index*** (-2) | 0.00575 | 27 | 0.00367 | 54 | 0.00021 | 285 |
| Rainfall** (-3) | 0.00563 | 28 | 0.00368 | 53 | 0.00124 | 163 |
| Rainfall*** (-1) | 0.00559 | 29 | 0.00415 | 22 | 0.00006 | 319 |
| Solar radiation** | 0.00554 | 30 | 0.00388 | 32 | 0.00021 | 289 |
