# Supplementary material for: Mapping trends in insecticide resistance phenotypes in African malaria vectors
Source: PLoS Biol. 2020 Jun 25;18(6):e3000633. doi: 10.1371/journal.pbio.3000633 (PMC7316233; doi:10.1371/journal.pbio.3000633)
Supplement: S6 Table — The 30 variables that were most highly ranked by XGB are shown. Definitions of each predictor variable are given in S9 Table. Variable name suffixes (-1), (-2) and (-3) denote time lags of 1, 2, and 3 years, respectively. One, two and three asterisks denote the first, second, and third principal component, respectively, for variables available on a monthly time step. XGB, extreme gradient boosting model. (DOCX) [file pbio.3000633.s017.docx]

| **Predictor variable** | **Variable importance** | | | | | |
| --- | --- | --- | --- | --- | --- | --- |
|  | **XGB** | **Rank** | **RF** | **Rank** | **BGAM** | **Rank** |
| Rainfall* (-3) | 0.06202 | 1 | 0.00575 | 6 | 0.04008 | 5 |
| Maximum diurnal temperature difference ** | 0.03069 | 2 | 0.00423 | 24 | 0.00832 | 23 |
| Vegetation index** (-3) | 0.02288 | 3 | 0.00429 | 22 | 0.00021 | 261 |
| Solar radiation** | 0.02094 | 4 | 0.00598 | 5 | 0.00029 | 243 |
| Maximum diurnal temperature difference*** (-1) | 0.01920 | 5 | 0.00550 | 8 | 0.00470 | 49 |
| Surface wetness** (-3) | 0.01736 | 6 | 0.00438 | 21 | 0.00215 | 106 |
| Wind speed* | 0.01524 | 7 | 0.00573 | 7 | 0.00510 | 41 |
| ITN coverage (-3) | 0.01404 | 8 | 0.00707 | 1 | 0.01427 | 12 |
| Maximum day time temperature** (-3) | 0.01250 | 9 | 0.00451 | 17 | 0.00435 | 58 |
| ITN coverage (-2) | 0.01120 | 10 | 0.00668 | 3 | 0.00725 | 27 |
| ITN coverage (-1) | 0.01014 | 11 | 0.00668 | 4 | 0.00493 | 43 |
| Maximum vegetation index** (-3) | 0.01004 | 12 | 0.00318 | 95 | 0.00004 | 294 |
| Maximum vegetation index** (-2) | 0.00951 | 13 | 0.00364 | 51 | 0.00089 | 160 |
| Sedimentation | 0.00927 | 14 | 0.00542 | 9 | 0.01774 | 11 |
| Pyrethroid IRS (-2) | 0.00903 | 15 | 0.00510 | 13 | 0.00453 | 52 |
| Surface wetness** (-1) | 0.00814 | 16 | 0.00355 | 60 | 0.00648 | 32 |
| Minimum surface wetness** | 0.00786 | 17 | 0.00682 | 20 | 0.00224 | 101 |
| Surface wetness** | 0.00766 | 18 | 0.00251 | 204 | 0.01004 | 17 |
| Rainfall intensity** (-1) | 0.00744 | 19 | 0.00534 | 10 | 0.00368 | 65 |
| Vegetation index*** (-1) | 0.00743 | 20 | 0.00486 | 16 | 0.00458 | 51 |
| Rainfall*** | 0.00722 | 21 | 0.00411 | 28 | 0.00267 | 86 |
| Pyrethroid IRS (-1) | 0.00657 | 22 | 0.00533 | 11 | 0.00275 | 82 |
| ITN coverage | 0.00575 | 23 | 0.00491 | 15 | 0.02751 | 7 |
| Vegetation index*** (-3) | 0.00569 | 24 | 0.00369 | 48 | 0.00256 | 91 |
| Rainfall*** (-1) | 0.00549 | 25 | 0.00492 | 14 | 0.00106 | 149 |
| Maximum diurnal temperature difference*** | 0.00547 | 26 | 0.00259 | 188 | 0.00767 | 26 |
| Area of non-food crops | 0.00535 | 27 | 0.00346 | 67 | 0.00525 | 39 |
| Rainfall intensity** (-3) | 0.00505 | 28 | 0.00514 | 12 | 0.00574 | 36 |
| Vegetation index** | 0.00481 | 29 | 0.00266 | 170 | 0.00023 | 254 |
| Pyrethroid IRS (-3) | 0.00481 | 30 | 0.00417 | 27 | 0.00037 | 231 |
