# Supplementary material for: Mapping trends in insecticide resistance phenotypes in African malaria vectors
Source: PLoS Biol. 2020 Jun 25;18(6):e3000633. doi: 10.1371/journal.pbio.3000633 (PMC7316233; doi:10.1371/journal.pbio.3000633)
Supplement: S7 Table — (DOCX) [file pbio.3000633.s018.docx]

| **West Region** | | **East Region** | **Combined** |
| --- | --- | --- | --- |
| Deltamethrin | 1356 | 1249 | 2605 |
| Permethrin | 843 | 611 | 1454 |
| Lambdacyhalothrin | 316 | 363 | 679 |
| Alphacypermethrin | 252 | 84 | 336 |
| DDT | 748 | 601 | 1349 |
| *Vsgc* allele frequency | 215 | 101 | 316 |
|  | **3730** | **3009** | **6739** |
