# Supplementary material for: Mapping trends in insecticide resistance phenotypes in African malaria vectors
Source: PLoS Biol. 2020 Jun 25;18(6):e3000633. doi: 10.1371/journal.pbio.3000633 (PMC7316233; doi:10.1371/journal.pbio.3000633)
Supplement: S8 Table — (DOCX) [file pbio.3000633.s019.docx]

|  | **2005** | **2006** | **2007** | **2008** | **2009** | **2010** | **2011** | **2012** | **2013** | **2014** | **2015** | **2016** | **2017** |
| --- | --- | --- | --- | --- | --- | --- | --- | --- | --- | --- | --- | --- | --- |
| Pyrethroids | 95 | 79 | 72 | 183 | 373 | 367 | 551 | 473 | 568 | 634 | 593 | 537 | 549 |
| DDT | 58 | 34 | 35 | 80 | 172 | 140 | 106 | 111 | 92 | 141 | 149 | 124 | 107 |
