# Supplementary material for: Mapping trends in insecticide resistance phenotypes in African malaria vectors
Source: PLoS Biol. 2020 Jun 25;18(6):e3000633. doi: 10.1371/journal.pbio.3000633 (PMC7316233; doi:10.1371/journal.pbio.3000633)
Supplement: S9 Table — If the data layer was obtained from an online repository, the URL and date accessed are given. If the data layer has a citation, then this is given. (DOCX) [file pbio.3000633.s020.docx]

| **Short name** | **Description** | **Temporal resolution** | **Lags** | **URL** | **Date accessed** | **Citation** |
| --- | --- | --- | --- | --- | --- | --- |
| **Insecticide-based malaria intervention coverage** | | | | | | |
| ITN coverage | ITN coverage (proportion of people protected) | annual | 0, 1, 2, 3 years | https://map.ox.ac.uk/explorer/#/ | n/a | 39 |
| Pyrethroid IRS | Coverage of indoor residual spraying with pyrethroids (proportion of households sprayed) | annual | 0, 1, 2, 3 years | n/a | n/a | 40 |
| Organochlorine IRS | Coverage of indoor residual spraying with DDT (proportion of households sprayed) | annual | 0, 1, 2, 3 years | n/a | n/a | 40 |
| *Anopheles gambiae* complex species | | | | | | |
| Arabiensis vs gambiae/coluzzii | Proportional abundance of *An. arabiensis* to *An. coluzzii*/*gambiae* | static | n/a | n/a | n/a | 41 |
| **Processes associated with pesticide fate in the environment** | | | | | | |
| Leaching | Infiltration and percolation of rain or irrigation water to deeper groundwater layers. | n/a | n/a | n/a | n/a | 42 |
| Surface runoff generation | Mechanisms involved in the generation of surface runoff of rain or irrigation water. | n/a | n/a | n/a | n/a | 42 |
| Surface runoff transfer | Transfer of rain or irrigation water overland to other streams or surface water. | n/a | n/a | n/a | n/a | 42 |
| Surface runoff accumulation | Streams or surface waters where rain or irrigation water accumulates. | n/a | n/a | n/a | n/a | 42 |
| Sedimentation | Soil particles in suspension settle out of fluid, water in this instance, and come to rest. | n/a | n/a | n/a | n/a | 42 |
| Soil storage and filtering capacity | Capacity of a soil to store and filter chemical substances. | n/a | n/a | n/a | n/a | 42 |
| Volatilization^†^ | Chemical substances convert from a liquid or solid state to a gaseous or vapour state. | monthly | n/a | n/a | n/a | 42 |
| **Crop and livestock variables** | | | | | | |
| Cropland percentage | Proportion of the pixel area covered by annual crops (temporary crops with harvest period or bare soil) | annual | 0, 1, 2, 3 years | <https://modis.gsfc.nasa.gov/data/dataprod/mod12.php> | 30 July 2018 | 43 |
| Cropland-natural vegetation percentage | Proportion of the pixel area covered by a mosaic of annual crops and natural vegetation (mosaic of cropland, forest, shrubland or grassland) | annual | 0, 1, 2, 3 years | https://modis.gsfc.nasa.gov/data/dataprod/mod12.php | 30 July 2018 | 43 |
| Rice^*^ | Rice production in 2005 (metric tonne) | static | n/a | http://harvestchoice.org/data/rice_p | 8 Feb 2018 | 44 |
| Cotton | Cotton production in 2005 (metric tonne) | static | n/a | https://harvestchoice.org/data/cott_p | 8 Feb 2018 | 45 |
| Sugar cane | Sugar cane production in 2005 (metric tonne) | static | n/a | https://harvestchoice.org/data/sugc_p | 8 Feb 2018 | 46 |
| Maize^*^ | Maize production in 2005 (metric tonne) | static | n/a | https://harvestchoice.org/data/maiz_p | 8 Feb 2018 | 47 |
| Non-food^*^ | Non-food crop production in 2005 (metric tonne) | static | n/a | https://harvestchoice.org/data/area_nonf | 12 Feb 2018 | 48 |
| Banana^*^ | Banana and plantain production in 2005 (metric tonne) | static | n/a | https://harvestchoice.org/data/bapl_p | 8 Feb 2018 | 49 |
| Barley | Barley production in 2005 (metric tonne) | static | n/a | https://harvestchoice.org/data/barl_p | 8 Feb 2018 | 50 |
| Bean | Bean production in 2005 (metric tonne) | static | n/a | https://harvestchoice.org/data/bean_p | 8 Feb 2018 | 51 |
| Cassava | Cassava production in 2005 (metric tonne) | static | n/a | https://harvestchoice.org/data/cass_p | 8 Feb 2018 | 52 |
| Cereal^*^ | Cereal production in 2005 (metric tonne) | static | n/a | https://harvestchoice.org/data/cere_p | 8 Feb 2018 | 53 |
| Chickpea | Chickpea production in 2005 (metric tonne) | static | n/a | https://harvestchoice.org/data/chic_p | 8 Feb 2018 | 54 |
| Cocoa^*^ | Cocoa production in 2005 (metric tonne) | static | n/a | https://harvestchoice.org/data/coco_p | 8 Feb 2018 | 55 |
| Coconut | Coconut production in 2005 (metric tonne) | static | n/a | https://harvestchoice.org/data/cnut_p | 8 Feb 2018 | 56 |
| Coffee | Coffee production in 2005 (metric tonne) | static | n/a | https://harvestchoice.org/data/coff_p | 8 Feb 2018 | 57 |
| Cowpea | Cowpea production in 2005 (metric tonne) | static | n/a | https://harvestchoice.org/data/cowp_p | 8 Feb 2018 | 58 |
| Groundnut | Groundnut production in 2005 (metric tonne) | static | n/a | https://harvestchoice.org/data/grou_p | 8 Feb 2018 | 59 |
| Lentil | Lentil production in 2005 (metric tonne) | static | n/a | https://harvestchoice.org/data/lent_p | 12 Feb 2018 | 60 |
| Millet | Millet production in 2005 (metric tonne) | static | n/a | https://harvestchoice.org/data/mill_p | 12 Feb 2018 | 61 |
| Other cereals | Other cereals production in 2005 (metric tonne) | static | n/a | https://harvestchoice.org/data/ocer_p | 12 Feb 2018 | 62 |
| Other fibres | Other fibre crop production in 2005 (metric tonne) | static | n/a | https://harvestchoice.org/data/ofib_p | 12 Feb 2018 | 63 |
| Other oils^*^ | Other oil crop production in 2005 (metric tonne) | static | n/a | https://harvestchoice.org/data/ooil_p | 12 Feb 2018 | 64 |
| Other pulses | Other pulses production in 2005 (metric tonne) | static | n/a | https://harvestchoice.org/data/opul_p | 22 Apr 2018 | 65 |
| Other root crops^*^ | Other roots and tubers crop production in 2005 (metric tonne) | static | n/a | https://harvestchoice.org/data/orts_p | 12 Feb 2018 | 66 |
| Palmoil^*^ | Palm oil production in 2005 (metric tonne) | static | n/a | https://harvestchoice.org/data/oilp_p | 12 Feb 2018 | 67 |
| Pigeonpea | Pigeonpea production in 2005 (metric tonne) | static | n/a | https://harvestchoice.org/data/pige_p | 12 Feb 2018 | 68 |
| Potato^*^ | Potato production in 2005 (metric tonne) | static | n/a | https://harvestchoice.org/data/pota_p | 12 Feb 2018 | 69 |
| Pulses^*^ | Pulses production in 2005 (metric tonne) | static | n/a | https://harvestchoice.org/data/puls_p | 12 Feb 2018 | 70 |
| Rapeseed | Rapeseed production in 2005 (metric tonne) | static | n/a | https://harvestchoice.org/data/rape_p | 12 Feb 2018 | 71 |
| Sesame | Sesame production in 2005 (metric tonne) | static | n/a | https://harvestchoice.org/data/sesa_p | 12 Feb 2018 | 72 |
| Sorghum | Sorghum production in 2005 (metric tonne) | static | n/a | https://harvestchoice.org/data/sorg_p | 22 Apr 2018 | 73 |
| Soybean^*^ | Soybean production in 2005 (metric tonne) | static | n/a | https://harvestchoice.org/data/soyb_p | 12 Feb 2018 | 74 |
| Sunflower | Sunflower production in 2005 (metric tonne) | static | n/a | https://harvestchoice.org/data/sunf_p | 12 Feb 2018 | 75 |
| Sweet potato^*^ | Sweet potato production in 2005 (metric tonne) | static | n/a | https://harvestchoice.org/data/swpo_p | 12 Feb 2018 | 76 |
| Temperate fruit | Temperate fruits production in 2005 (metric tonne) | static | n/a | https://harvestchoice.org/data/temf_p | 12 Feb 2018 | 77 |
| Tea | Tea production in 20005 (metric tonne) | static | n/a | https://harvestchoice.org/data/teas_p | 12 Feb 2018 | 78 |
| Tobacco^*^ | Tobacco production in 2005 (metric tonne) | static | n/a | https://harvestchoice.org/data/toba_p | 12 Feb 2018 | 79 |
| Tropical fruit | Tropical fruits production in 2005 (metric tonne) | static | n/a | https://harvestchoice.org/data/trof_p | 12 Feb 2018 | 80 |
| Vegetables | Vegetables production in 2005 (metric tonne) | static | n/a | https://harvestchoice.org/data/vege_p | 12 Feb 2018 | 81 |
| Wheat | Wheat production in 2005 (metric tonne) | static | n/a | https://harvestchoice.org/data/whea_p | 12 Feb 2018 | 82 |
| Yams^*^ | Yams production in 2005 (metric tonne) | static | n/a | https://harvestchoice.org/data/yams_p | 12 Feb 2018 | 83 |
| Irrigated area^*^ | Areas equipped for irrigation | static | n/a | http://www.fao.org/nr/water/aquastat/irrigationmap/index10.stm | 28 Aug 2018 | 84 |
| Harvested area | Harvested area of all crops in 2005 | static | n/a | http://harvestchoice.org/data/area_crop | 27 Feb 2018 | 85 |
| Grazing prod. | Grazing production for bovines in 2000 (kg/ha) | static | n/a | https://harvestchoice.org/data/bv_graz | 30 May 2018 | 86 |
| Stover prod. ^*^ | Stover production for bovines in 2000 (kg/ha) | static | n/a | https://harvestchoice.org/data/bv_stov | 30 May 2018 | 87 |
| Cattle | Cattle density in 2005 (head/km^2^) | static | n/a | https://harvestchoice.org/data/ad05_catt | 30 May 2018 | 88 |
| Sheep^*^ | Sheep density in 2005 (head/km^2^) | static | n/a | https://harvestchoice.org/data/ad05_shee | 30 May 2018 | 89 |
| Goats | Goat density in 2005 (head/km^2^) | static | n/a | https://harvestchoice.org/data/ad05_goat | 30 May 2018 | 90 |
| Pigs^*^ | Pig population in 2005 (head) | static | n/a | https://harvestchoice.org/data/an05_pig | 30 May 2018 | 91 |
| Chickens^*^ | Poultry density in 2005 (head/km^2^) | static | n/a | https://harvestchoice.org/data/ad05_chic | 30 May 2018 | 92 |
| Livestock density | Livestock density in 2005 (LU/km^2^) | static | n/a | https://harvestchoice.org/data/ad05_lu | 30 May 2018 | 93 |
| Other land cover variables | | | | | | |
| Evergreen broadleaf percentage | Proportional cover of evergreen broadleaf forest (>60% land covered with broadleaf vegetation of height >2m and canopy never without green foliage) | annual | 0, 1, 2, 3 years | https://modis.gsfc.nasa.gov/data/dataprod/mod12.php | 30 Jul 2018 | 43 |
| Mixed forest percentage | Proportional cover of mixed forest (>60% land covered with vegetation of height >2m and mosaic of the four forest types) | annual | 0, 1, 2, 3 years | https://modis.gsfc.nasa.gov/data/dataprod/mod12.php | 30 Jul 2018 | 43 |
| Closed shrubland percentage | Proportional cover of closed shrublands (woody vegetation <2m tall with canopy cover >60% of area) | annual | 0, 1, 2, 3 years | https://modis.gsfc.nasa.gov/data/dataprod/mod12.php | 23 March 2018 | 43 |
| Open shrubland percentage | Proportional cover of open shrublands (vegetation <2m tall and shrub canopy cover >60% of area) | annual | 0, 1, 2, 3 years | https://modis.gsfc.nasa.gov/data/dataprod/mod12.php | 23 March 2018 | 43 |
| Woody savanna percentage | Proportional cover of woody savanna (trees 30-60% and understory vegetation) | annual | 0, 1, 2, 3 years | https://modis.gsfc.nasa.gov/data/dataprod/mod12.php | 23 March 2018 | 43 |
| Savanna percentage | Proportional cover of savanna (trees 10-30% and understory vegetation) | annual | 0, 1, 2, 3 years | https://modis.gsfc.nasa.gov/data/dataprod/mod12.php | 30 Jul 2018 | 43 |
| Grassland percentage | Proportional cover of grasslands (herbaceous cover with trees/shrubs <10%) | annual | 0, 1, 2, 3 years | https://modis.gsfc.nasa.gov/data/dataprod/mod12.php | 23 March 2018 | 43 |
| Permanent wetland percentage | Proportional cover of permanent wetlands (a permanent mixture of water and vegetation over extensive areas) | annual | 0, 1, 2, 3 years | https://modis.gsfc.nasa.gov/data/dataprod/mod12.php | 23 March 2018 | 43 |
| Barren and sparsely populated area percentage | Proportional cover of barren and sparsely populated areas (land with exposed soil, sand or rocks, with <10% vegetation cover at any time) | annual | 0, 1, 2, 3 years | https://modis.gsfc.nasa.gov/data/dataprod/mod12.php | 2 Aug 2018 | 43 |
| **Other variables** | | | | | | |
| Population density | Human population size (No. persons/pixel) | annual | 0, 1, 2, 3 years | <https://www.worldpop.org/geodata/listing?id=17> | n/a | 94 |
| Drainage class | Classification for the rate at which water infiltrates into the soil. | n/a | n/a | http://data2.isric.org/geonetwork/srv/api/records/953d0964-6746-489a-a8d1-f188595516a9 | 9 Nov 2018 | 95 |
| Soil moisture | Moisture content of a soil (%). | n/a | n/a | https://smap.jpl.nasa.gov/data/ | 1 Dec 2018 | 96 |
| Bedrock | Depth at which bedrock occurs (cm) | n/a | n/a | https://files.isric.org/soilgrids/data/recent/ | 1 Dec 2018 | 97 |
| Flow accumulation | Based on the digital elevation model a map on flow accumulation was created. | n/a | n/a | https://hydrosheds.org/ | 26 July 2018 | 98 |
| Slope | Slope of the land (°) | n/a | n/a | https://cgiarcsi.community/data/srtm-90m-digital-elevation-database-v4-1/ | 23 March 2018 | 99 |
| Soil depth | Depth of the soil layer (cm) | n/a | n/a | n/a | n/a | 100 |
| Rainfall erosivity factor | Factor that indicates the kinetic energy of raindrop’s impact and the rate of associated runoff. | n/a | n/a | https://esdac.jrc.ec.europa.eu/content/global-rainfall-erosivity | 4 Sep 2018 | 101 |
| Slope-length factor | Factor that describes the effect of slope steepness and the impact of slope length. | n/a | n/a | n/a | n/a | 42 |
| Erosion^*^ | Total detachment and removal of soil material by water (t/ha/yr). | n/a | n/a | n/a | n/a | 42 |
| Cation exchange capacity | Cation exchange capacity of a soil is a measure for the amount of cations that can retain on soil particle surfaces (cmol_c_/kg) | n/a | n/a | https://files.isric.org/soilgrids/data/recent/ | 21 Feb 2018 | 97 |
| Clay content | Percent of clay particles (<2μm) in the soil (%). | n/a | n/a | https://files.isric.org/soilgrids/data/recent/ | 21 Feb 2018 | 97 |
| Soil organic carbon | Organic carbon content in the soil (g/kg) | n/a | n/a | https://files.isric.org/soilgrids/data/recent/ | 21 Feb 2018 | 97 |
| Soil pH | Soil pH is a measure of acidity or alkalinity of a soil. | n/a | n/a | https://files.isric.org/soilgrids/data/recent/ | 6 Feb 2018 | 97 |
| GUF | Binary map of urban areas in 2011 (areas featuring man-made building structures with a vertical component) | static | no | <https://www.dlr.de/eoc/en/desktopdefault.aspx/tabid-9628/16557_read-40454/> | 7 Feb 2017 | 102 |
| **Climatic variables** | | | | | | |
| Solar rad. ^†^ | Solar radiation (kJ/m^2^/day) | monthly | n/a | <http://worldclim.org/version2> | 4 Sep 2018 | 103 |
| Wind speed^†^ | Long-term (1970-2000) average wind speed (m/s) | monthly | n/a | http://worldclim.org/version2 | 16 Jan 2018 | 103 |
| Relative humidity | Average relative humidity (ratio of the partial pressure of water vapour to the equilibrium vapour pressure of water) between 2015 and 2018 (%) | static | n/a | <https://developers.google.com/earth-engine/datasets/catalog/NOAA_GFS0P25> | 3 Dec 2018 | 104 |
| Vegetation index max^†^ | Maximum enhanced vegetation index is a measure of greenness reflectance of the land surface | annual  monthly | 0, 1, 2, 3 years | https://lpdaac.usgs.gov/products/mcd43d6*2-4*v006/ | 17 Sep 2018 | 105 |
| Vegetation index mean^†^ | Mean enhanced vegetation index is a measure of greenness reflectance of the land surface | annual  monthly | 0, 1, 2, 3 years | https://lpdaac.usgs.gov/products/mcd43d6*2-4*v006/ | 17 Sep 2018 | 105 |
| Vegetation index min^†^ | Minimum enhanced vegetation index is a measure of greenness reflectance of the land surface | annual  monthly | 0, 1, 2, 3 years | https://lpdaac.usgs.gov/products/mcd43d6*2-4*v006/ | 17 Sep 2018 | 105 |
| Land surface temp. day max^†^ | Maximum land surface daytime temperature (°C) gap-filled from the source. | annual monthly | 0, 1, 2, 3 years | <https://lpdaac.usgs.gov/products/mod11a2v006/> | 9 Oct 2018 | 106 |
| Land surface temp. day mean^†^ | Mean land surface daytime temperature (°C) gap-filled from the source. | annual monthly | 0, 1, 2, 3 years | https://lpdaac.usgs.gov/products/mod11a2v006/ | 9 Oct 2018 | 106 |
| Land surface temp. day min^†^ | Minimum land surface daytime temperature (°C) gap-filled from the source. | annual monthly | 0, 1, 2, 3 years | https://lpdaac.usgs.gov/products/mod11a2v006/ | 9 Oct 2018 | 106 |
| Land surface temp. diurnal diff max^†^ | Maximum difference between corresponding surface daytime temperature and surface night-time temperature images (°C) | annual monthly | 0, 1, 2, 3 years | https://lpdaac.usgs.gov/products/mod11a2v006/ | 4 Oct 2018 | 106 |
| Land surface temp. diurnal diff mean^†^ | Mean difference between corresponding surface daytime temperature and surface night-time temperature images (°C) | annual monthly | 0, 1, 2, 3 years | https://lpdaac.usgs.gov/products/mod11a2v006/ | 4 Oct 2018 | 106 |
| Land surface temp. diurnal diff min^†^ | Minimum difference between corresponding surface daytime temperature and surface night-time temperature images (°C) | annual monthly | 0, 1, 2, 3 years | https://lpdaac.usgs.gov/products/mod11a2v006/ | 4 Oct 2018 | 106 |
| Land surface temp. night max^†^ | Maximum land surface night-time temperature (°C) | annual monthly | 0, 1, 2, 3 years | https://lpdaac.usgs.gov/products/mod11a2v006/ | 5 Oct 2018 | 106 |
| Land surface temp. night mean^†^ | Mean land surface night-time temperature (°C) | annual monthly | 0, 1, 2, 3 years | https://lpdaac.usgs.gov/products/mod11a2v006/ | 5 Oct 2018 | 106 |
| Land surface temp. night min^†^ | Minimum land surface night-time temperature (°C) | annual monthly | 0, 1, 2, 3 years | https://lpdaac.usgs.gov/products/mod11a2v006/ | 5 Oct 2018 | 106 |
| Rainfall^†^ | Total precipitation (mm) | annual monthly | 0, 1, 2, 3 years | http://chg.geog.ucsb.edu/data/chirps/#_Data | 27 Nov 2017 | 107 |
| Rainfall Intensity^†^ | Average precipitation intensity (total precipitation/No. precipitation days) (mm) | annual monthly | 0, 1, 2, 3 years | http://chg.geog.ucsb.edu/data/chirps/#_Data | 11 Dec 2018 | 107 |
| Bare surface moisture max^†^ | Maximum values for a measure of moisture on bare surfaces (TCB, variation in soil background reflectance) | annual monthly | 0, 1, 2, 3 years | https://lpdaac.usgs.gov/products/mcd43d6*2-4*v006/ | 6 Dec 2018 | 108 |
| Bare surface moisture mean^†^ | Mean values for a measure of moisture on bare surfaces (TCB, variation in soil background reflectance) | annual monthly | 0, 1, 2, 3 years | https://lpdaac.usgs.gov/products/mcd43d6*2-4*v006/ | 6 Dec 2018 | 108 |
| Bare surface moisture min^†^ | Minimum values for a measure of moisture on bare surfaces (TCB, variation in soil background reflectance) | annual monthly | 0, 1, 2, 3 years | https://lpdaac.usgs.gov/products/mcd43d6*2-4*v006/ | 6 Dec 2018 | 108 |
| Surface wetness max^†^ | Maximum values for a measure of surface moisture (TCW, variation in the vigour of green vegetation) | annual monthly | 0, 1, 2, 3 years | https://lpdaac.usgs.gov/products/mcd43d6*2-4*v006/ | 3 Oct 2018 | 108 |
| Surface wetness mean^†^ | Mean values for a measure of surface moisture (TCW, variation in the vigour of green vegetation) | annual monthly | 0, 1, 2, 3 years | https://lpdaac.usgs.gov/products/mcd43d6*2-4*v006/ | 3 Oct 2018 | 108 |
| Surface wetness min^†^ | Minimum values for a measure of surface moisture (TCW, variation in the vigour of green vegetation) | annual monthly | 0, 1, 2, 3 years | https://lpdaac.usgs.gov/products/mcd43d6*2-4*v006/ | 3 Oct 2018 | 108 |
| Potential evapotranspiration max | Max. potential evapotranspiration (water vapour flux under ideal conditions) between 1950 and 2000 (mm) | static | n/a | <https://cgiarcsi.community/data/global-aridity-and-pet-database/> | 5 Feb 2015 | 109 |
| Potential evapotranspiration mean | Mean potential evapotranspiration (water vapour flux under ideal conditions) between 1950 and 2000 (mm) | static | n/a | <https://cgiarcsi.community/data/global-aridity-and-pet-database/> | 5 Feb 2015 | 109 |
| Potential evapotranspiration min | Min. potential evapotranspiration (water vapour flux under ideal conditions) between 1950 and 2000 (mm) | static | n/a | <https://cgiarcsi.community/data/global-aridity-and-pet-database/> | 5 Feb 2015 | 109 |
| Potential evapotranspiration st.dev. | Standard deviation of the potential evapotranspiration (water vapour flux under ideal conditions) between 1950 and 2000 (mm) | static | n/a | <https://cgiarcsi.community/data/global-aridity-and-pet-database/> | 5 Feb 2015 | 109 |
| Elevation | Elevation measured using the hydrologically conditioned Digital Elevation Model (m) | n/a | n/a | https://hydrosheds.org/ | 27 March 2018 | 98 |
| Distance water | Distance to water, including rivers, surface waters and oceans (m) | n/a | n/a | https://hydrosheds.org/ | 5 Nov 2015 | 98 |

† Conducted a principal component analysis on variables for each month and selected the top three principal components.

* For the boosted generalized additive model (BGAM), the variable was discretised according to a set of bins describing consecutive, non-overlapping intervals of the variable.
