# Supplementary material for: Mapping trends in insecticide resistance phenotypes in African malaria vectors
Source: PLoS Biol. 2020 Jun 25;18(6):e3000633. doi: 10.1371/journal.pbio.3000633 (PMC7316233; doi:10.1371/journal.pbio.3000633)
Supplement: S10 Table — (DOCX) [file pbio.3000633.s021.docx]

**Table S10**. *l0* models and parameters

| **Model** | **Parameter** | | | |
| --- | --- | --- | --- | --- |
|  | Name | Description | Value | Tuned? |
| XGB | max_depth | maximum depth of a tree | 8 | Yes |
|  | nrounds | number of trees | 12000 | Yes |
|  | eta | learning rate | 0.001 | Yes |
|  | gamma | minimum loss reduction required to make a further partition | 0.5 | Yes |
|  | min_child_weight | minimum sum of instance weight needed in a child | 7 | Yes |
|  | colsample_by_tree | subsample ratio of columns when constructing a tree | 0.7 | Yes |
|  | subsample | subsample ratio of the training instance | 0.7 | Yes |
|  | rate_drop | proportion of trees dropped each iteration | 0.001 | Yes |
|  | lambda | regularization parameter | 1 | Yes |
| RF | mtry | number of variables randomly sampled at each split | 150 | Yes |
|  | ntree | number of trees | 1001 | No |
|  | col_sample_rate_per_tree | rate of randomly sampling features used for each tree | 0.8 | Yes |
|  | node_size | minimum number of observations per leaf | 20 | Yes |
| BGAM | mstop | final number of iterations | 80000 | Yes |
|  | degree | degrees of freedom of the base learners | 1 | No |
|  | nu | Shrinkage parameter | 0.4 | Yes |
