# Supplementary material for: Mapping trends in insecticide resistance phenotypes in African malaria vectors
Source: PLoS Biol. 2020 Jun 25;18(6):e3000633. doi: 10.1371/journal.pbio.3000633 (PMC7316233; doi:10.1371/journal.pbio.3000633)
Supplement: S1 Text — (PDF) [file pbio.3000633.s022.pdf]

SUPPLEMENTARY MATERIAL

"Mapping trends in insecticide resistance phenotypes in African malaria vectors"

Hancock PA, Hendriks CJM, Tangena J-A, Gibson H, Hemingway J, Coleman M, Gething PW, Bhatt S, Moyes CL

S1. Spatiotemporal trends in resistance to pyrethroids: permethrin, lambda-cyhalothrin and alpha-cypethrin.......2

S2. Fitted parameter values of the geostatistical models.......2

S3. Posterior predictive checks.......2

S4. Influential predictor variables.......3

S5. Correlations across influential predictor variables.......3

S6. Bioassay data: selection criteria and spatio-temporal distribution.......3

S7. Description of potential predictor variables.......4

S8. Spatiotemporal Bayesian statistical models.......5

S9. Implementing Gaussian process stacked generalization.......6

S1. Spatiotemporal trends in resistance to pyrethroids: permethrin, lambda-cyhalothrin and alpha-cypethrin

We developed predictive maps of the mean prevalence of resistance to four pyrethriods: deltamethrin, permethrin, lambda-cyhalothrin and alpha-cypermethrin. Results for deltamethrin are reported in the main text, and similar spatiotemporal trends were found in the predicted prevalence of resistance to permethrin (Fig. S1), lambda-cyhalothrin (Fig. S2) and alpha-cypermethrin (Fig. S3). Our results show consistently higher prevalence of resistance to some pyrethroid insecticides compared to others (for example the predicted prevalence of resistance to permethrin is higher than that for deltamethrin). It is not possible to determine whether this is due to differences in resistance to these insecticides or to differences in the calibration of the diagnostic doses, especially as a representative range of laboratory strains is not always available for the calibration process [1].

S2. Fitted parameter values of the geostatistical models

The fitted parameters of each Bayesian Gaussian process regression meta-model are shown in Table S1. The variance parameters σ^2_ω and σ^2 are in the units of the (IHS and empirical logit) transformed susceptibility test observations. Range is in the units of the unit sphere.

S3. Posterior predictive checks

S3.1. Probability integral transform histograms

To assess how well the observations are represented by the predictive distributions given by the Bayesian Gaussian process regression models, w used histograms of the probability integral transform (PIT) values. Before any data are seen a minimally-informative prior will typically produce prior predictive distribution that is vastly over-dispersed relative to the observed data. The ambition of a Bayesian spatial model is that as data i made available the hierarchical structure will facilitate a sharing of information such that the posterior predictive for data unseen (or those withhel during cross-validation) will be close to the true data generating distribution. In this ideal case, the PIT diagnostic has a standard uniform distributio [2]. The R-INLA package (http://www.r-inla.org) provides approximate cross-validated PIT values where the PIT value for observation *i* is adjusted t omit the contribution of the ith observation to the posterior predictive distribution [3]. For the model for pyrethroids, we found that the cross-validate PIT values were approximately uniformly distributed for the models for both the west (Fig. S4A) and east (Fig. S4B) regions.

S3.2. K-fold out-of-sample validation

The predictive performance of our models was assessed using 10-fold out-of-sample validation (Tables S2 and S3 and Fig. S5). Of the three constituen models, the extreme gradient boosting (XGB) model showed the best predictive performance for both pyrethroid and DDT resistance, and had the highest weight in the model ensemble (Table S4). We also performed 10-fold out-of-sample validation to assess the accuracy of the credible intervals of the posterior distributions of predicted mean mortality. We generated 1000 posterior predictions for each data point $g_A(\mathbf{s}_i,t)$ (eqn 1 in main text) using the inla.posterior.sample function available in R-INLA (www.r-inla.org)[4], and adding uncorrelated random noise, e_A. Credible intervals based on the quantiles of these posterior predictions were found to accurately predict the error associated with withheld data (Fig. S6). The credible intervals associated with the predicted mean prevalence of resistance to deltamethrin across the west and east regions are presented in the main text. The

corresponding credible intervals associated with the predicted mean prevalence of resistance to DDT (Fig. S7) also show strong spatial heterogeneity, with particularly high prediction uncertainty in the north west part of the east region.

S4. Influential predictor variables

The variable importance measures produced by each model show that variables describing ITN usage are relatively important predictors in all thre[illegible] of the constituent models of the ensemble (Tables S5 and S6). For the west region variables relating to ITN coverage are ranked in the top two mos[illegible] important for all three models, and in the top twelve most important for all three models for the east region.

S5. Correlations across influential predictor variables

Correlations between predictor variables make it difficult to interpreting variable importance measures [5], because the influence of a particula[illegible] variable can be masked by that of other variables with which it is correlated. In our analysis, ITN coverage had the highest variable importance valu[illegible] in the model developed for the west African region. ITN coverage is not highly correlated with other variables (Fig. S10), with the exception o[illegible] potential evapotranspiriation (PET). This indicates that ITN coverage is likely to be an influential predictor of insecticide resistance in the west regior[illegible] Rainfall had the highest variable importance value in the model developed for the east African region. Rainfall is highly correlated with other highl[illegible] ranked climate variables (Fig. S11) including rainfall intensity, temperature variation, relative humidity, vegetation index and surface wetness. Due t[illegible] these high interactions with other climate variables, it is not possible to conclude that rainfall is important to predicting insecticide resistanc[illegible] independently of these other variables. We can, however, conclude that climate variables are likely to be more important than ITN coverage i[illegible] predicting insecticide resistance in the east region, because there are not strong correlations between ITN coverage and climate variables (Fig. S11).

S6. Bioassay data: selection criteria and spatio-temporal distribution

Our insecticide resistance bioassay data set includes information about the mosquitoes tested, the number of mosquitoes in the sample, the sampl[illegible] collection and the bioassay conditions and protocol [6]. Some of this information was used to select a subset of records for inclusion in our stud[illegible] including the mosquito sample identification data (available at either the genus, complex or species level), the collection site location, the bioassa[illegible] protocol, the insecticide tested and whether a synergist was used, and the insecticide concentration and exposure period. We only included bioassay[illegible] that were conducted over the period 2005-2017 on samples collected within two separate spatial regions: a rectangular region in West Africa tha[illegible] extends from 0°N to 17.1°N and -18°W to 19°W, and a rectangular region in East Africa that extends from -28°N to 16.5°N and 24.5°W to 51.5°W (Fi[illegible] 1). Only bioassay results for which the sample collection location was assigned a point coordinate were included, noting that for these bioassays th[illegible] collection area is less than 25km^2 [6]. Further, we consider only bioassays conducted on mosquito samples that were identified as belonging to the *A[illegible] gambiae* complex. The majority of these studies (~80%) did not identify the individual species within the *An. gambiae* complex to which the sample[illegible] mosquitoes belonged, therefore our study does not account for differences in insecticide resistance phenotypes that can occur across these siblin[illegible] species [7, 8, 9, 10, 11, 12, 13, 14, 15, 16]. We did not included any bioassay results for which the sample tested was biased with respect to the original sampl[illegible] collected from the field (see Hancock et al. [17]).

We included bioassays conducted using either pyrethroid insecticides or DDT. For pyrethroids this included four insecticides: deltamethri[illegible] permethrin, λ-cyhalothrin and α-cypermethrin. For organochlorines, only bioassays conducted using DDT were included. Bioassays for which th[illegible] insecticide concentration or the exposure time differed from that defined by the standard WHO insecticide susceptibility bioassay protocol [1] wer[illegible] excluded from the analysis. Also excluded were any bioassays performed on a sample of less than 20 mosquitoes and any bioassays that use[illegible]

synergists. The final data set included a total of 6,423 data points that met these criteria (see Table S7 for a breakdown by insecticide type and across the east and west regions and Table S8 for the number of records per year).

S7. Description of potential predictor variables

All predictor variables included in our models are detailed in Table S9. Variables describing vector control interventions include a yearly measure o the coverage of ITNs and IRS. Values of ITN coverage were obtained from maps that estimate the proportion of the population that were protected b ITNs for each year [18, 19]. Values of IRS coverage for each insecticide type were obtained from maps that estimate the proportion of households spraye with each insecticide class within each year [20]. We note that the data provide an IRS coverage value for each 5km^2 pixel but the spatial unit of th original coverage values ranges from third-order administrative divisions to national-level, and varies across countries and years. The relativ abundance of *An. arabiensis* vs *An. coluzzii*/*gambiae* was obtained from a model of the baseline contemporary relative abundance of these species a every ~5km pixel across Africa that used field data collected from 1985 to 2015 [21].

Data on the use of insecticides in agriculture across the two study regions were not available. Variables representing agricultural land use and th environmental fate of insecticides were used instead. Variables describing agricultural land use included data on the production of crops and livestock Variables relating to crop production included measures of the production of 30 individual crop types (in tonnes), obtained from HarvestChoice [2 (Table S9). These crop production layers were only available for a single year (2005). In our models we assumed temporally static production for al crops, estimated using the 2005 layers. However, we included a variable containing a yearly measure of the proportional area covered by crops (of an type), obtained from MODerate Resolution Imaging Spectroradiometer (MODIS) MCD12Q1 IBGP landcover data sets [23]. Variables relating to livestoc production included measures of the densities (head/km^2) of 5 different livestock types (cattle, chicken, sheep, goats and pigs) as well as a variabl estimating the proportional area used for grazing animals. All livestock layers were obtained from HarvestChoice, and were again only available fo the year 2005. In our models we used the 2005 layers to estimate livestock densities for all years.

Variables associated with the environmental fate of insecticides included maps of seven key processes: leaching, surface runoff generation, surfac runoff transfer, surface runoff accumulation, sedimentation, soil storage and filtering capacity, volatilization [24]. The first six processes wer constructed using geological data such as soil types and topography, and the most contemporary data available were used. Volitalization wa estimated for each month and we performed a Principal Components Analysis (PCA) across months using the R package RStoolBox and used the to three principal components as variables. In addition, we included a set of geological variables that were used in constructing the above five layers These included distance to water [25], variation in soil thermal capacity [26], soil pH [27], organic carbon content [27], clay content [27], and the Cation Exchang Capacity (CEC) [27].

Our set of variables describing climate processes included measures of day time and night time temperature, diurnal temperature variation, vegetation index (EVI), and two measures of surface wetness (TCW and TCB), all of which were obtained from MODIS data sets [23] plus rainfall an rainfall intensity obtained from the Climate Hazards Group InfraRed Precipitation with Station data [28]. Gaps in the MODIS data caused by cloud cove were filled using a spatio-temporal gap-filling algorithm [29]. The data provide estimates for each month and year, so for each variable we performed PCA across the monthly data for each year and used the top three principal components to describe that variable for each year. We also include

variables describing potential evapotranspiration (PET), relative humidity and wind speed, all of which were based on averages across years [30]. In addition, monthly average estimates of solar radiation were included (Table S9).

Finally, we included variables describing annual variation in land cover, using 14 of the land cover classes defined by MODIS [31] that applied to the tw[illegible] study regions. A static variable describing the proportional area devoted to urban land use, obtained from the Global Urban Footprint measurement [32], was also included. In addition, we included a variable that provides annual estimates of human population density at every ~5km pixel, obtaine[illegible] from the WorldPop data set [33].

S8. Spatiotemporal Bayesian statistical models

We defined a Bayesian hierarchical formulation of our model of the proportional mortality records for bioassays conducted using an insecticide type [illegible] as follows:

$$g_A(\mathbf{s}_i,t) \mid f_A(\mathbf{s}_i,t),\theta_A \sim N(\mathbf{w}_A\mathbf{M}_A + \mathbf{A}f_A(\mathbf{s}_i,t),\sigma_A^2) \qquad \text{(S1a)}$$

where

$$Cov(\omega_A(\mathbf{s}_i,t),\omega_A(\mathbf{s}_j,t')) = \begin{cases} 0, & t \neq t' \\ \sigma_{\omega_A}^2 C_A(h), & t = t' \end{cases} \qquad \text{(S1b)}$$

and

$$\theta_A \sim \boldsymbol{\pi}_A \qquad \text{(S1c)}$$

where $\boldsymbol{\pi}_A$ is a vector of prior probability distributions for the hyperparameters $\theta_A = [\mathbf{w}_A,\sigma_A,\sigma_{\omega_A},\kappa_A,\phi_A]$ (see below for their specification) and $f_A(\mathbf{s}_i,t$[illegible] is a spatio-temporal Gaussian Markov Random Field (GMRF;). $\mathbf{w}_A$ is a vector containing the weights of each model in the ensemble (see the Method[illegible] section of the main text), σ_A is the standard deviation of the observation noise, respectively (also defined in the Methods section of the main text[illegible] $\sigma_{\omega_A},\kappa_A$ and ϕ_A are parameters of the GMRF (see below), and $\mathbf{A}$ is a sparse observation matrix that maps the GMRF to function evaluations at loca[illegible] observations. The spatio-temporal GMRF, $f_A(\mathbf{s}_i,t)$, is composed of a spatial GMRF that evolves according to a first order temporal auto-regressiv[illegible] process (AR(1)) with correlation ϕ_A. The innovation noise $\omega_A(\mathbf{s}_i,t)$ is a temporally uncorrelated Gaussian process with variance $\sigma_{\omega_A}^2$ and a spatia[illegible] correlation structure $C_A(h)$ that is constant in time and defines the spatial dependence structure of the observations [34]. Our spatial GMRF mode[illegible] follows the Integrated Nested Laplace Approximation (INLA) approach [35], whereby we assume that $C_A(h)$ is a Matérn covariance function. I[illegible] developing the INLA approach, Lindgren et al. [35] used the Matérn covariance function to define an approximation to a GMRF using stochastic partia[illegible] differential equations. The Matérn covariance function $C_A(h)$ depends on the spatial separation distance between two points, $h = \|\mathbf{s}_i - \mathbf{s}_j\|$ as well as [illegible] scale parameter κ_A and a fixed smoothness parameter ν which is set to 1. The spatial process is therefore assumed to be second-order stationary an[illegible] isotropic.

We used a PC precision prior [36] for the standard deviations σ_A and σ_{ω_A} setting the upper limit U = 5 and the probability α =0.01. Our prior for κ_A is given by setting a PC prior on the range parameter [37] setting the lower limit U=0.0003 and the probability α =0.01, noting that we define spatia locations using a 3D Cartesian coordinate system. Our prior for ϕ_A is a PC prior for the correlation parameter in a first order autoregressive (AR(1) model [37] where we set the upper limit U=0.5 and the probability α =0.5. For the weights $\mathbf{w}_A$ we use a constrained linear effect model, specified by usin the "clinear" model in R-INLA (www.r-inla.org)[4]. We constrained each weight to be within the range [0,1].
We tried alternative models for the data generating distribution (eqn S1a), including a binomial model, which represents the numbers of dead an alive mosquitoes from the susceptibility tests as a dichotomous random variable. We chose a Gaussian model for the (empirical logit and IH transformed) data because the binomial model under-estimated the data variance, producing U-shaped PIT histograms, and gave inferior out-o sample predictive performance as assessed by K-fold out-of-sample validation.

S9. Implementing Gaussian process stacked generalization

1. Data preparation

For predictor variables for which data is available on a monthly time step (Table S9), principal component analyses were performed on the sets o twelve variables containing the monthly data for each year in the time period. The top three principle components for each year were included a predictor variables in each of the level 0 machine learning models. The lags for these sets of principle components (including one, two and three yea lags) were also calculated and included as predictor variables, consistent with our treatment of the other dynamic predictor variables that vary over yearly time step.
Further manipulation of the predictor variable data depended on the type of level 0 model. For the extreme gradient boosting and random fores methods, each environmental predictor variable was normalised across the values corresponding to all response observations according to:

$$x_{i,new} = \frac{x_i - x_{\min}}{x_{\max} - x_{\min}} \quad \text{(S2)}$$

where x_i is the variable's original value, $x_{i,new}$ is the variable's transformed (normalised) value, and $x_{\min}$ and $x_{\max}$ are the variable's minimum an maximum values, respectively.
For the boosted generalized additive model some of the predictor variables were discretised across a discrete set of evenly spaced bins (Table S9). Th variables that were transformed in this way showed a long-tailed distribution with sparse data coverage across the variable's range, and th transformation was found to improve model performance with respect to out-of-sample extrapolation. All variables were then normalized accordin to eqn S2.

2. Tuning the parameters of the machine learning models

For each machine learning model, parameter tuning was performed using K-fold out-of-sample validation based on a set of K training and validatio subsets. Predictive performance was evaluated on a separate test data subset. The test set was selected by randomly sampling approximately ten pe cent of the records from the full data set without replacement. The training and validation folds were then selected from the remaining data records b random sampling without replacement. For each model, Table S10 shows the chosen values for each parameter. In applying the extreme gradien boosting method we used the DART boosting methodology to avoid over-specialization [38].

3. Performing stacked generalization with K-fold cross validation

Below we describe our procedure for applying stacked generalization using multiple level 0 models (indexed by p=1...M) and a single level 1 mode[illegible] We perform K-fold cross validation to assess the predictive performance of the stacked generalization procedure, fitting the stacked model to a serie[illegible] of K training sets and generating out-of-sample predictions for the corresponding K testing sets.

Step 1: A series of training and test sets was created by were subdividing the full set of n data records into K (=10) training and test sets using randon[illegible] sampling without replacement.

Step 2: Each of the K training sets was further subdivided into J (=10) training and validation sets using random sampling without replacement. Th[illegible] level 0 models (parameterised by their respective tuning sets; Table S10) were fitted to each of the J training sets and the fitted models were used t[illegible] generate out-of-sample predictions for the respective J validation sets. The combined set of out-of-sample predictions generated by the pth level [illegible] model across all J validation sets are denoted $\tilde{g}^{K}_{A,q,p}$ where A denotes the insecticide type and q=1...n_K, where n_K is the number of records in the K^{t}[illegible] training set. Thus across all J validation sets there is one out-of-sample prediction for every record in the Kth training set.

Step 3: Each of the level 0 models were fitted to the full set of records in the Kth training set and the fitted models were used to generate predictions fo[illegible] each of the respective K testing sets. These sets of predictions are denoted $\breve{g}^{K}_{A,q,r}$ where r=1...m_K, and m_K is the number of records in the Kth test set.

Step 4: For each of the K training sets, the level 1 Gaussian process regression model (eqn 1 in the main text) is then fit to all observations in the K^{t}[illegible] training set, $g_A(\mathbf{s}_k, t)$, with the elements of the design matrix $\mathbf{M}^{A}_{s_i,t}$ set to the out-of-sample predictions for each observation given by each of the M leve[illegible] 0 models i.e. $M_{q,p} = \tilde{g}_{A,q,p}$. The fitted level 1 model is then used to generate the final set of out-of-sample predictions for each of the K test sets by settin[illegible] the elements of $\mathbf{M}^{A}_{s_i,t}$ to the level 0 predictions of the records in the Kth test set, $\breve{g}_{A,q,r}$. Using R-INLA to fit the Gaussian process regression model, th[illegible] combined data and validation stacks for the Kth validation fold that are passed to the R-INLA model have the following format:

$$
\begin{array}{llll}
g_{A,1} & \tilde{g}^{K}_{A,1,1} & \cdots & \tilde{g}^{K}_{A,1,M} \\
\vdots & & & \\
g_{A,n_K} & \tilde{g}^{K}_{A,n_K,1} & \cdots & \tilde{g}^{K}_{A,n_K,M} \\
NA & \breve{g}^{K}_{A,n_K+1,1} & \cdots & \breve{g}^{K}_{A,n_K+1,M} \\
\vdots & & & \\
NA & \breve{g}^{K}_{A,n_K+m_K,1} & \cdots & \breve{g}^{K}_{A,n_K+m_K,M}
\end{array}
$$

where the first column contains the response data values and the remaining columns are modelled as fixed effects.

REFERENCES

1. World Health Organization. Test procedures for insecticide resistance monitoring in malaria vectors: bio-efficacy and persistance of insecticides on treated surfaces. (ed^(eds). WHO (1998).
2. Czado C, Gneiting T, Held L. Predictive Model Assessment for Count Data. *Biometrics* **65**, 1254-1261 (2009).
3. Held L, Schrodle B, Rue H. *Posterior and Cross-validatory Predictive Checks: A Comparison of MCMC and INLA* (2010).
4. Rue H, Held L. *Gaussian Markov Random Fields: Theory and Applications*. Chapman & Hall.
5. Strobl C, Boulesteix AL, Kneib T, Augustin T, Zeileis A. Conditional variable importance for random forests. *Bmc Bioinformatics* **9**, (2008).
6. Moyes CL*, et al.* Analysis-ready datasets for insecticide resistance phenotype and genotype frequency in African malaria vectors. *Scientific Data* **6**, 121 (2019).
7. Antonio-Nkondjio C*, et al.* Review of the evolution of insecticide resistance in main malaria vectors in Cameroon from 1990 to 2017. *Parasites & Vectors* **10**, (2017).
8. Awolola TS, Oduola OA, Strode C, Koekemoer LL, Brooke B, Ranson H. Evidence of multiple pyrethroid resistance mechanisms in the malaria vector Anopheles gambiae sensu stricto from Nigeria. *Transactions of the Royal Society of Tropical Medicine and Hygiene* **103**, 1139-1145 (2009).
9. Brooke BD*, et al.* Bioassay and biochemical analyses of insecticide resistance in southern African Anopheles funestus (Diptera : Culicidae). *Bulletin of Entomological Research* **91**, 265-272 (2001).
10. Nardini L, Christian RN, Coetzer N, Ranson H, Coetzee M, Koekemoer LL. Detoxification enzymes associated with insecticide resistance in laboratory strains of *Anopheles arabiensis* of different geographic origin. *Parasites & Vectors* **5**, (2012)
11. Nardini L*, et al.* Malaria vectors in the Democratic Republic of the Congo: the mechanisms that confer insecticide resistance in Anopheles gambiae and Anopheles funestus. *Malaria Journal* **16**, (2017).
12. Pinto J*, et al.* Multiple Origins of Knockdown Resistance Mutations in the Afrotropical Mosquito Vector Anopheles gambiae. *Plos One* **2**, (2007).
13. Riveron JM*, et al.* Directionally selected cytochrome P450 alleles are driving the spread of pyrethroid resistance in the major malaria vector *Anopheles funestus*. *Proceedings of the National Academy of Sciences of the United States of America* **110**, 252-257 (2013).
14. Sangba MLO*, et al.* Evidence of multiple insecticide resistance mechanisms in *Anopheles gambiae* populations in Bangui, Central African Republic. *Parasites & Vectors* **10**, (2017).
15. Santolamazza F*, et al.* Distribution of knock-down resistance mutations in Anopheles gambiae molecular forms in west and west-central Africa. *Malaria Journal* **7**, (2008).
16. Yahouedo GA*, et al.* Contributions of cuticle permeability and enzyme detoxification to pyrethroid resistance in the major malaria vector *Anopheles gambiae*. *Scientific Reports* **7**, (2017).
17. Hancock PA*, et al.* Associated patterns of insecticide resistance in field populations of malaria vectors across Africa. *Proceedings of the National Academy of Sciences of the United States of America* **115**, 5938-5943 (2018).
18. Bhatt S*, et al.* Coverage and system efficiencies of insecticide-treated nets in Africa from 2000 to 2017. *Elife* **4**, (2015).
19. Weiss DJ*, et al.* The global landscape of *Plasmodium falciparum* prevalence, incidence and mortality 2000-2017. *The Lancet* **accepted**, (2019).

20. Tangena J-A, *et al.* Indoor residual spraying for malaria control in Sub-Saharan Africa 1997 to 2017: an adjusted retrospective analysis. Available at SSRN: https://ssrn.com/abstract=tbc (2019).
21. Sinka ME, *et al.* Modelling the relative abundance of the primary African vectors of malaria before and after the implementation of indoor, i nsecticide-based vector control. *Malaria Journal* **15**, (2016).
22. You L, Wood-Sichra U, Fritz S, Guo Z, See L, Koo J. Spatial production allocation model (SPAM) 2005 v2.0. (ed^(eds). mapspam.info.
23. Friedl M, Sulla-Menashe D. MCD12Q1 MODIS/Terra+Aqua Land Cover Type Yearly L3 Global 500m SIN Grid V006. (ed^(eds). NASA EOSDIS Land Processes DAAC (2015).
24. Hendriks CJM, *et al.* Mapping geospatial processes affecting the environmental fate of agricultural pesticides in Africa. *EarthArXiv*, https://doi.org/10.31223/osf.io/db31222qz (2019).
25. Lehner B, Verdin K, Jarvis A. New global hydrography derived from spaceborne elevation data. *Eosk Trans Am Geophys Union* **89**, 93-94 (2008).
26. Global Soil Data Task Group. Global gridded surfaces of selected soil characteristics (IGBP-DIS). (ed^(eds). ORNL DAAC (2000).
27. Hengl T, *et al.* SoilGrids250m: Global gridded soil information based on machine learning. *Plos One* **12**, (2017).
28. Funk C, *et al.* The climate hazards infrared precipitation with stations - a new environmental record for monitoring extremes. *Scientific Data* **2**.
29. Weiss DJ, Atkinson PM, Bhatt S, Mappin B, Hay SI, Gething PW. An effective approach for gap-filling continental scale remotely sensed time-series. *ISPRS Journal of Photogrammetry and Remote Sensing* **98**, 106-118 (2014).
30. Trabucco A, Zomer RJ. Global Aridity Index (Global-Aridity) and Global Potential Evapo-Transpiration (Global-PET) Geospatial Database. (ed^(eds). CGIAR-CSI GeoPortal (2009).
31. Sulla-Menashe D, Gray JM, Abercrombie SP, Friedl MA. Hierarchical mapping of annual global land cover 2001 to present: The MODIS Collection 6 Land Cover product. *Remote Sensing of Environment* **222**, 183-194 (2019).
32. Esch T, *et al.* Breaking new ground in mapping human settlements from space - The Global Urban Footprint. *ISPRS Journal of Photogrammetry and Remote Sensing* **134**, 30-42 (2017).
33. Tatem AJ. WorldPop, open data for spatial demography. *Scientific Data* **4**, (2017).
34. Cameletti M, Lindgren F, Simpson D, Rue H. Spatio-temporal modeling of particulate matter concentration through the SPDE approach. *Asta-Advances in Statistical Analysis* **97**, 109-131 (2013).
35. Lindgren F, Rue H, Lindstrom J. An explicit link between Gaussian fields and Gaussian Markov random fields: the stochastic partial differential equation approach. *Journal of the Royal Statistical Society Series B-Statistical Methodology* **73**, 423-498 (2011).
36. Simpson D, Rue H, Riebler A, Martins TG, Sorbye SH. Penalising Model Component Complexity: A Principled, Practical Approach to Constructing Priors. *Statistical Science* **32**, 1-28 (2017).
37. Fuglstad G-A, Simpson D, Lindgren F, Rue H. Constructing Priors that Penalize the Complexity of Gaussian Random Fields. *Journal of the American Statistical Association* **114**, 445-452 (2019).
38. Rashmi K, Gilad-Bachrach R. DART: Dropouts meet Multiple Additive Regression Trees. *Proceedings of the Eighteenth International Conference on Artificial Intelligence and Statistics, PMLR* **38**, 489-497 (2015).
39. Bhatt S, Weiss DJ, Mappin B, Dalrymple U, Cameron E, Bisanzio D, Smith DL, Moyes CL, Tatem AJ, Lynch M, Fergus CA, Yukich J, Bennett A, Eisele TP, Kolaczinski J, Cibulskis RE, Hay SI and Gething PW (2015) Coverage and system efficiencies of insecticide-treated nets in Africa from 2000 to 2017. *eLife*, e09672.

40. Tangena JA, Hendriks CMJ, Devine M, Tammaro M, Trett AE, Williams I, DePina AJ, Sisay A, Herizo R, Kafy HT, Chizema E, Were A, Rozier J, Coleman M, Moyes CL (2019) Indoor residual spraying for malaria control in Sub-Saharan Africa 1997 to 2017; an adjusted retrospective analysis. Available at SSRN: https://ssrn.com/abstract=tbc.
41. Sinka ME, Golding N, Massey NC, Wiebe A, Huang Z, Hay SI, Moyes CL (2016) Modelling the relative abundance of the primary vectors of malari[illegible] in Africa and the effect of indoor insecticide-based interventions. *Malaria Journal*, 15: 142.
42. Hendriks C, Gibson H, Trett A, Python A, Weiss D, Vrieling A, Weiss D., Coleman M, Gething P, Hancock P, Moyes C (2019) Mapping geospatial processes affecting the environmental fate of agricultural pesticides in Africa. EarthArXiv, 10.31223/osf.io/db2qz.
43. MODIS/Terra and Aqua Combined Land Cover Type Yearly Global 500 m SIN Grid - MCD12Q1.006. Friedl, M., Sulla-Menashe, D. (2015). MCD12Q1 MODIS/Terra+Aqua Land Cover Type Yearly L3 Global 500m SIN Grid V006 [Data set]. NASA EOSDIS Land Processes DAAC. doi: 10.5067/MODIS/MCD12Q1.006.
44. HarvestChoice, 2015. "Rice Production (mt, 2005)." International Food Policy Research Institute, Washington, DC., and University of Minnesota St. Paul, MN. Available online at http://harvestchoice.org/data/rice_p.
45. HarvestChoice, 2015. "Cotton Production (mt, 2005)." International Food Policy Research Institute, Washington, DC., and University of Minnesota, St. Paul, MN. Available online at http://harvestchoice.org/data/cott_p.
46. HarvestChoice, 2015. "Sugar cane Production (mt, 2005)." International Food Policy Research Institute, Washington, DC., and University of Minnesota, St. Paul, MN. Available online at http://harvestchoice.org/data/sugc_p.
47. HarvestChoice, 2015. "Maize Production (mt, 2005)." International Food Policy Research Institute, Washington, DC., and University of Minnesota, St. Paul, MN. Available online at http://harvestchoice.org/data/maiz_p.
48. HarvestChoice, 2015. "Non-food Production (mt, 2005)." International Food Policy Research Institute, Washington, DC., and University of Minnesota, St. Paul, MN. Available online at http://harvestchoice.org/data/nonf_p.
49. HarvestChoice, 2015. "Bananas & Plantains Yield (mt, 2005)." International Food Policy Research Institute, Washington, DC., and University of Minnesota, St. Paul, MN. Available online at http://harvestchoice.org/data/bapl_p
50. HarvestChoice, 2015. "Barley Production (mt, 2005)." International Food Policy Research Institute, Washington, DC., and University of Minnesota, St. Paul, MN. available online at http://harvestchoice.org/data/barl_p.
51. HarvestChoice, 2015. "Bean Production (mt, 2005)." International Food Policy Research Institute, Washington, DC., and University of Minnesota, St. Paul, MN. Available online at http://harvestchoice.org/data/bean_p.
52. HarvestChoice, 2015. "Cassava Production (mt, 2005)." International Food Policy Research Institute, Washington, DC., and University of Minnesota, St. Paul, MN. Available online at http://harvestchoice.org/data/cass_p.
53. HarvestChoice, 2015. "Cereals Production (mt, 2005)." International Food Policy Research Institute, Washington, DC., and University of Minnesota, St. Paul, MN. Available online at http://harvestchoice.org/data/cere_p.
54. HarvestChoice, 2015. "Chickpea Production (mt, 2005)." International Food Policy Research Institute, Washington, DC., and University of Minnesota, St. Paul, MN. Available online at http://harvestchoice.org/data/chic_p.
55. HarvestChoice, 2015. "Cocoa Production (mt, 2005)." International Food Policy Research Institute, Washington, DC., and University of Minnesota, St. Paul, MN. Available online at http://harvestchoice.org/data/coco_p.
56. HarvestChoice, 2015. "Coconut Production (mt, 2005)." International Food Policy Research Institute, Washington, DC., and University of Minnesota, St. Paul, MN. Available online at http://harvestchoice.org/data/cnut_p.

57. HarvestChoice, 2015. " Coffee Production (mt, 2005)." International Food Policy Research Institute, Washington, DC., and University of Minnesota, St. Paul, MN. Available online at http://harvestchoice.org/data/coff_p.
58. HarvestChoice, 2015. "Cowpea Production (mt, 2005)." International Food Policy Research Institute, Washington, DC., and University of Minnesota, St. Paul, MN. Available online at http://harvestchoice.org/data/cowp_p.
59. HarvestChoice, 2015. "Groundnut Production (mt, 2005)." International Food Policy Research Institute, Washington, DC., and University of Minnesota, St. Paul, MN. Available online at http://harvestchoice.org/data/grou_p.
60. HarvestChoice, 2015. "Lentil Production (mt, 2005)." International Food Policy Research Institute, Washington, DC., and University of Minnesota, St. Paul, MN. Available online at http://harvestchoice.org/data/lent_p.
61. HarvestChoice, 2015. "Millet Production (mt, 2005)." International Food Policy Research Institute, Washington, DC., and University of Minnesota, St. Paul, MN. Available online at http://harvestchoice.org/data/mill_p.
62. HarvestChoice, 2015. "Other Cereals Production (mt, 2005)." International Food Policy Research Institute, Washington, DC., and University of Minnesota, St. Paul, MN. Available online at http://harvestchoice.org/data/ocer_p.
63. HarvestChoice, 2015. "Other Fibers Production (mt, 2005)." International Food Policy Research Institute, Washington, DC., and University of Minnesota, St. Paul, MN. Available online at http://harvestchoice.org/data/ofib_p.
64. HarvestChoice, 2015. "Other Oils Production (mt, 2005)." International Food Policy Research Institute, Washington, DC., and University of Minnesota, St. Paul, MN. Available online at http://harvestchoice.org/data/ooil_p.
65. HarvestChoice, 2015. "Other Pulses Production (mt, 2005)." International Food Policy Research Institute, Washington, DC., and University of Minnesota, St. Paul, MN. Available online at http://harvestchoice.org/data/opul_p.
66. HarvestChoice, 2015. "Other Roots and Tubers Production (mt, 2005)." International Food Policy Research Institute, Washington, DC., and University of Minnesota, St. Paul, MN. Available online at http://harvestchoice.org/data/orts_p.
67. HarvestChoice, 2015. "Oil Palm Production (mt, 2005)." International Food Policy Research Institute, Washington, DC., and University of Minnesota, St. Paul, MN. Available online at http://harvestchoice.org/data/oilp_p.
68. HarvestChoice, 2015. "Pigeonpea Production (mt, 2005)." International Food Policy Research Institute, Washington, DC., and University of Minnesota, St. Paul, MN. Available online at http://harvestchoice.org/data/pige_p.
69. HarvestChoice, 2015. "Potato Production (mt, 2005)." International Food Policy Research Institute, Washington, DC., and University of Minnesota, St. Paul, MN. Available online at http://harvestchoice.org/data/pot_p.
70. HarvestChoice, 2015. "Pulses Production (mt, 2005)." International Food Policy Research Institute, Washington, DC., and University of Minnesota, St. Paul, MN. Available online at http://harvestchoice.org/data/puls_p.
71. HarvestChoice, 2015. "Rapeseed Production (mt, 2005)." International Food Policy Research Institute, Washington, DC., and University of Minnesota, St. Paul, MN. Available online at http://harvestchoice.org/data/rape_p.
72. HarvestChoice, 2015. "Sesame Production (mt, 2005)." International Food Policy Research Institute, Washington, DC., and University of Minnesota, St. Paul, MN. Available online at http://harvestchoice.org/data/sesa_p.
73. HarvestChoice, 2015. "Sorghum Production (mt, 2005)." International Food Policy Research Institute, Washington, DC., and University of Minnesota, St. Paul, MN. Available online at http://harvestchoice.org/data/sorg_p.
74. HarvestChoice, 2015. "Soybean Production (mt, 2005)." International Food Policy Research Institute, Washington, DC., and University of Minnesota, St. Paul, MN. Available online at http://harvestchoice.org/data/soyb_p.

75. HarvestChoice, 2015. "Sunflower Production (mt, 2005)." International Food Policy Research Institute, Washington, DC., and University of Minnesota, St. Paul, MN. Available online at http://harvestchoice.org/data/sunf_p.
76. HarvestChoice, 2015. "Sweet Potato Production (mt, 2005)." International Food Policy Research Institute, Washington, DC., and University of Minnesota, St. Paul, MN. Available online at http://harvestchoice.org/data/swpo_p.
77. HarvestChoice, 2015. "Temperate Fruits Production (mt, 2005)." International Food Policy Research Institute, Washington, DC., and University of Minnesota, St. Paul, MN. Available online at http://harvestchoice.org/data/temf_p.
78. HarvestChoice, 2015. "Tea Production (mt, 2005)." International Food Policy Research Institute, Washington, DC., and University of Minnesota, St. Paul, MN. Available online at http://harvestchoice.org/data/teas_p.
79. HarvestChoice, 2015. "Tobacco Production (mt, 2005)." International Food Policy Research Institute, Washington, DC., and University of Minnesota, St. Paul, MN. Available online at http://harvestchoice.org/data/toba_p.
80. HarvestChoice, 2015. "Tropical Fruits Production (mt, 2005)." International Food Policy Research Institute, Washington, DC., and University of Minnesota, St. Paul, MN. Available online at http://harvestchoice.org/data/trof_p.
81. HarvestChoice, 2015. "Vegetable Production (mt, 2005)." International Food Policy Research Institute, Washington, DC., and University of Minnesota, St. Paul, MN. Available online at http://harvestchoice.org/data/vege_p.
82. HarvestChoice, 2015. "Wheat Production (mt, 2005)." International Food Policy Research Institute, Washington, DC., and University of Minnesota, St. Paul, MN. Available online at http://harvestchoice.org/data/whea_p.
83. HarvestChoice, 2015. "Yam Production (mt, 2005)." International Food Policy Research Institute, Washington, DC., and University of Minnesota St. Paul, MN. Available online at http://harvestchoice.org/data/yams_p.
84. Stefan Siebert, Verena Henrich, Karen Frenken and Jacob Burke (2013). Global Map of Irrigation Areas version 5. Rheinische Friedrich-Wilhelms-University, Bonn, Germany / Food and Agriculture Organization of the United Nations, Rome, Italy.
85. HarvestChoice, 2015. "Crops Harvested Area (ha, 2005)." International Food Policy Research Institute, Washington, DC., and University of Minnesota, St. Paul, MN. Available online at http://harvestchoice.org/data/area_crop.
86. HarvestChoice, 2015. "Bovine Grazing Production (kg/ha, 2000)." International Food Policy Research Institute, Washington, DC., and University of Minnesota, St. Paul, MN. Available online at http://harvestchoice.org/data/bv_graz.
87. HarvestChoice, 2015. "Bovine Stover Production (kg/ha, 2000)." International Food Policy Research Institute, Washington, DC., and University of Minnesota, St. Paul, MN. Available online at http://harvestchoice.org/data/bv_stov.
88. HarvestChoice, 2015. "Cattle Density (head/sq. km, 2005)." International Food Policy Research Institute, Washington, DC., and University of Minnesota, St. Paul, MN. Available online at http://harvestchoice.org/data/ad05_catt.
89. HarvestChoice, 2015. "Sheep Density (head/sq. km, 2005)." International Food Policy Research Institute, Washington, DC., and University of Minnesota, St. Paul, MN. Available online at http://harvestchoice.org/data/ad05_shee.
90. HarvestChoice, 2015. "Goat Density (head/sq. km, 2005)." International Food Policy Research Institute, Washington, DC., and University of Minnesota, St. Paul, MN. Available online at http://harvestchoice.org/data/ad05_goat.
91. HarvestChoice, 2015. "Pig Population (head, 2005)." International Food Policy Research Institute, Washington, DC., and University of Minnesota, St. Paul, MN. Available online at http://harvestchoice.org/data/an05_pig.
92. HarvestChoice, 2015. "Poultry Density (head/sq. km, 2005)." International Food Policy Research Institute, Washington, DC., and University of Minnesota, St. Paul, MN. Available online at http://harvestchoice.org/data/ad05_chic.

93. HarvestChoice, 2015. "Livestock Density (LU/sq. km, 2005)." International Food Policy Research Institute, Washington, DC., and University of Minnesota, St. Paul, MN. Available online at http://harvestchoice.org/data/ad05_lu.
94. Tatem AJ (2017) WorldPop, open data for spatial demography. *Scientific Data*, 4, 170004.
95. Hengl T, Heuvelink GBM, Kempen B, Leenaars JGB, Walsh MG, Shepherd KD, et al. Mapping Soil Properties of Africa at 250 m Resolution: Random Forests Significantly Improve Current Predictions. PLoS One. 2015; 25;10:e0125814.
96. Mladenova IE, Bolten JD, Crow WT, Anderson MC, Hain CR, Johnson DM, et al. Intercomparison of Soil Moisture, Evaporative Stress, and Vegetation Indices for Estimating Corn and Soybean Yields Over the U.S. IEEE J Sel Top Appl Earth Obs Remote Sens. 2017;10:1328–43.
97. Hengl T, Mendes de Jesus J, Heuvelink GBM, Ruiperez Gonzalez M, Kilibarda M, Blagotic A, et al. SoilGrids250m: Global gridded soil information based on machine learning. PLoS One. 2017;12:e0169748.
98. Lehner B, Verdin K, Jarvis A. New global hydrography derived from spaceborne elevation data. Eos, Trans Am Geophys Union. 2008;89:93–4.
99. Jarvis A, Reuter HI, Nelson A, Guevara E. Hole-filled SRTM for the globe Version 4. CGIAR-CSI SRTM 90m Database. 2008. http://srtm.csi.cgiar.org. Accessed 23 March 2018.
100. Stoorvogel JJ, Bakkenes M, Temme AJAM, Batjes NH, ten Brink BJE. S-World: A Global Soil Map for Environmental Modelling. L Degrad Dev. 2017;28:22–33.
101. Panagos P, Borrelli P, Meusburger K, Yu B, Klik A, Jae Lim K, et al. Global rainfall erosivity assessment based on high-temporal resolution rainfal records. Sci Rep. 2017;7:4175.
102. Esch, T., Heldens, W., Hirner, A., Keil, M., Marconcini, M., Roth, A., Zeidler, J., Dech, S., Strano, E. (2017): Breaking new ground in mapping human settlements from space – The Global Urban Footprint. ISPRS Journal of Photogrammetry and Remote Sensing 134 (2017) 30-42. https://doi.org/10.1016/ j.isprsjprs.2017.10.012
103. Fick SE, Hijmans RJ. WorldClim 2: new 1-km spatial resolution climate surfaces for global land areas. Int J Climatol. 2017;37:4302–15.
104. Earth Engine Data Catalog: GFS: Global Forecast System 384-Hour Predicted Atmosphere Data. https://developers.google.com/earth-engine/datasets/catalog/NOAA_GFS0P25. Accessed 3 Dec 2018.
105. Didan K, Munoz AB, Solano R, Huete A (2015) MODIS Vegetation Index User's Guide (MOD13 Series), University of Arizona, https://vip.arizona.edu/documents/MODIS/MODIS_VI_UsersGuide_June_2015_C6.pdf.
106. Wan, Z., S. Hook, G. Hulley. MOD11A2 MODIS/Terra Land Surface Temperature/Emissivity 8-Day L3 Global 1km SIN Grid V006. 2015, distributed by NASA EOSDIS Land Processes DAAC, https://doi.org/10.5067/MODIS/MOD11A2.006. https://lpdaac.usgs.gov/products/mcd43d62v006/
107. Funk, Chris, Pete Peterson, Martin Landsfeld, Diego Pedreros, James Verdin, Shraddhanand Shukla, Gregory Husak, James Rowland, Laura Harrison, Andrew Hoell & Joel Michaelsen."The climate hazards infrared precipitation with stations—a new environmental record for monitoring extremes". *Scientific Data* 2, 150066. doi:10.1038/sdata.2015.66 2015.
108. Lobser SE & Cohen WB (2007) MODIS tasselled cap: land cover characteristics expressed through transformed MODIS data, International Journal of Remote Sensing, 28:22, 5079-5101Trabucco A, Zomer RJ. Global Aridity Index (Global-Aridity) and Global Potential Evapo-Transpiration (Global-PET) Geospatial Database. CGIAR-CSI GeoPortal. 2009. https://cgiarcsi.community/data/global-aridity-and-pet-database/. Acccesed 21 Oct 2009.
109. Trabucco A, Zomer RJ. Global Aridity Index (Global-Aridity) and Global Potential Evapo-Transpiration (Global-PET) Geospatial Database. CGIAR-CSI GeoPortal. 2009. https://cgiarcsi.community/data/global-aridity-and-pet-database/. Acccesed 21 Oct 2009.
