# Supplementary material for: Mapping trends in insecticide resistance phenotypes in African malaria vectors
Source: PLoS Biol. 2020 Jun 25;18(6):e3000633. doi: 10.1371/journal.pbio.3000633 (PMC7316233; doi:10.1371/journal.pbio.3000633)
Supplement: Attachment — Submitted filename: Responses to reviewers.pdf [file pbio.3000633.s023.pdf]

Editor:

As you will read, the reviewers appreciated many aspects of your work and the importance of these maps. They also raise a number of points that will need to be addressed in a revision. Of particular note, we like the suggestion of Rev #2 to add the data of An.funestus resistance, if it is available. Rev #3 also raises a number of excellent points. We agree that these type of additional analyses would improve the manuscript, but do not consider them to be absolutely essential for a revision. We would like you to consider them and address them as best you can, providing clear explanation for those analyses you do not choose to include in the revision.

As detailed in our responses below, we have added results on *An. funestus* resistance to the manuscript in response to the comments of Reviewer 2, and we have explained that this data is insufficient to support geostatistical modelling analysis of the kind performed in this study. We have performed additional analyses which have been able to address some of the questions raised by Reviewer 3, and we have provided explanations for some suggestions that we were not able to address.

Reviewer 1:

Abstract.
Is it possible to quantify the "alarming increase", e.g. "since 2005 we have shown a X fold increase in malaria resistance across…"
We have adjusted the final sentence of the abstract to "Our maps show alarming increases in the prevalence of resistance to pyrethroids and DDT across Sub-Saharan Africa from 2005-2017, with mean mortality following insecticide exposure declining from almost 100% to less than 30% in some areas, as well as substantial spatial variation in resistance trends."

Introduction
Line 74 should be "after the introduction of these interventions".

Thank you, this has been corrected.

The passage from the paragraph in lines 63-83 to the paragraph in lines 85-98 is a bit abrupt. Is it possible to add a paragraph on how this problem (mapping insecticide resistance) has been dealt before? Also how current models fails in achieving what you have achieved.

We have added the following text to the Introduction (page 2, lines 67-75):

"Several studies have reported spatial variation in resistance within a country, but these studies haven't analysed the spatial trends behind this variation [11, 12]. In addition, time series of resistance data have shown changes over time at the location of sentinel sites [13, 14, 15, 16, 17, 18, 19, 20], however, only one study has investigated temporal trends across Africa [7]. This study generated a single trend over time for the whole continent, for each species and insecticide, and did not investigate differences in trends between locations. No previous study has analysed spatial variation across multiple countries or investigated spatio-temporal trends within these regions.

".

Results

If I was a general reader, I would found very difficult the interpretation of the RMSE for the comparison of the 4 models. First of all, in table S2 and S3 keep consistency with names (GAMB or BGAM). In terms of values it is not clear to me why you use different units between the main text and the supplementary. If necessary, please add a sentence in the main text.

We have corrected the Supplementary Material to refer to the boosted generalized additive model as BGAM instead of GAMB. We have now altered tables S2 and S3 to report the RMSE in terms of the transformed data and the un-transformed data.

If I compare figure 1 and 5, it seems to me that lines F,G,H,I and relative areas (figure 1) may not conclusive since uncertainty is close to 1 or very high (Figure 5). Please comment in the discussions or provide a bit more information.

We agree that predictive uncertainty is important and have reported it in our Results in reference to the trends at points F, G, H and I. At page 4 line 195, we state that "Across most of this area, mean mortality to deltamethrin in 2017 was below 0.5 (CI < 0.75)". We have also added a new Figure 7 (e.g. the trends for Ethiopia and Sudan). Here, the credible intervals account for the uncertainty in predicted mean mortality at each location within each country and, despite the relatively wide credible intervals in this region, we are able to quantify clear temporal trends.
The maximum width of the 95% credible intervals in the north east of Africa is close to 0.75. We note that this doesn't correspond to an uncertainty of 75%, because Bayesian credible intervals are measured by the quantiles of the posterior distribution, and there is greater credibility about values in the centre of the distribution than at the tails (provided the posterior is not completely flat).

In addition, looking at table S7 and S8 I speculate that uncertainty increases although samples are increasing too. Is this the effect of interventions that by fragmenting the "continuous" resistance, reduces the capacity of the spatio-temporal dependence to explain part of the variation?

A major reason why predictive uncertainty increases (credible intervals become wider) in more recent years compared to earlier years is because resistance is higher (proportional sample mortality values are lower). Proportional mortality values are much more variable when they are lower (i.e. well below 100%), whereas when a mosquito population is susceptible, mortality values are consistently high (close to 100%). There is therefore greater uncertainty in the mean value of resistance when resistance is high than when it is lower, and this means that credible intervals are wider in more recent years, even though the sample sizes are larger.

I found very interesting the importance of rainfall in your models, especially for

East Africa which was affected by two important droughts during your study period. Please can you add a comment on this?

We believe that it is important to be cautious about inferring causal relationships between resistance and the predictor variables included in our model. It is not possible with the type of data that we have (resistance surveillance data collected under widely varying conditions) and the modelling approach we use to make causal inferences. With this in mind, we agree that it is interesting to think about why climatological variables, such as rainfall, were influential predictors in our models.
We have added text to the Discussion (page 8, lines 375-380) explaining that variables such as climate processes that delineate broad spatial regions may possibly identify areas of similarity in resistance characteristics due to population structure. It is also possible that rainfall (and droughts) may have causal effects on resistance surveillance results, and humidity is known to have a substantial influence on susceptibility test results. Our analysis does not, however, allow us to test these hypotheses.

Methods
Variable importance. Instead of using different functions (and packages) to evaluate variable importance for each model, it would not be easier to just remove a variable at time and look at the RMSE?
There is a large body of literature comparing variable importance measures for particular modelling approaches, showing how biases can occur, particularly when there are correlations between predictor variables (e.g. Louppe et al. 2013, Advances in Neural Information Processing Systems). We use variable importance measures that are recommended in the literature, for example in the case of the random forest algorithm we use a measure based on permutation importance, rather than the Gini index which is more strongly biased (Strobl et al. 2007, Bioinformatics).

Reviewer 2:

The results for East Africa is not as convincing than the West Africa one, as the authors omit An. funestus resistance that has a major roles in the Southern part of the continent but is also in the past few years increasingly present in East Africa countries (Burundi, Tanzania, Uganda, Kenya). The An.funestus resistance data are also available and applying the model would provide important information that are missing in the actual paper especially as the models aimed to assist decision making in resistance management. The frequency of insecticide resistance in East Africa is not as widespread than in West Africa and resistance management in this area might be the most successful.

We fully agree that *An. funestus* is an important vector and resistance in *An. funestus* is an important topic. We have collated *An. funestus* data but unfortunately the data volumes are much lower than those available for *An. gambiae* s.l. Of the 796 pyrethroid and DDT susceptibility test data points available for the *An. funestus* subgroup, 692 meet our inclusion criteria of at least

20 mosquitoes tested from a single point location – compared to 6423 data points for *An. gambiae* s.l. (Moyes et al. 2019).

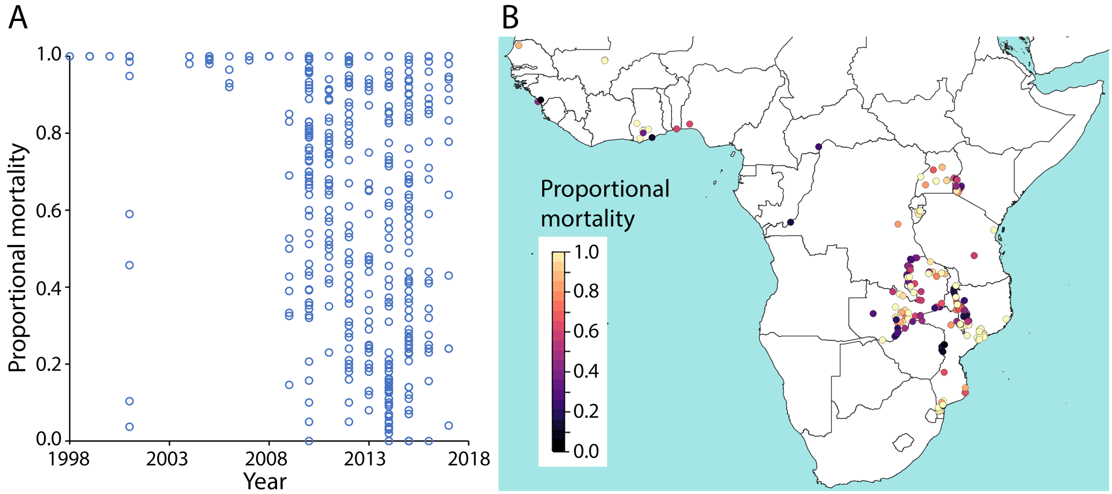

The figure above shows the data available for pyrethroid resistance from 1998 to 2018. There is insufficient data to robustly quantify trends in time and space in the way we have done for *An. gambiae* s.l., but we note that the data indicate higher levels of resistance in southeast Africa than we see for *An. gambiae* s.l. in this area, which backs up Reviewer #2's point. We have added the figure above to the Discussion (page 9) and text at lines 411-414 to highlight the observed higher levels of pyrethroid resistance in *An. funestus* in the south east.

The contribution of public health vs agriculture insecticides to selection of resistance is essential to device appropriate resistance management. One of the main limitation concerning the predictors is the lack of information on the insecticide/pesticide use for agriculture. The authors used different variables as a proxy, land use of crops and other land cover, fate of insecticides in the environment, etc. These variables correspond to more than half of the variables included in the model. Variables that cover the widest area modelled (ITN, Rainfall, temperature etc..) seems to be the highest contributors. I am therefore wondering if the apparent lower contribution of agriculture pesticide in An. gambiae insecticide resistance is due to the number of variables that have been included. Could the land use variables representing crops using pesticides the most, be pulled together as a single variable in the model? While I understand the difficulty to get the data on agriculture pesticide and find appropriate proxies, the authors should discuss, more in details, some of their findings and differences observed between West and East Africa. The model seems to suggest that agriculture pesticide use is a more important contributor in West than East Africa. Please comments.

We agree that the use of pesticides in agriculture is potentially an important influence on resistance in mosquito populations, and we have included numerous predictor variables into our model that describe patterns of agricultural land use and pesticide fate (Table S9). The machine learning modelling methodology that we employ allows for flexible, non-linear

interactions between the predictor variables and the response. These models link together the suite of variables relating to agricultural pesticide use and deposition, with the aim of capturing interactions between these variables that are influential to prediction of resistance trends.

It is important to be cautious about interpreting modelled relationships between predictor variables and insecticide resistance as causal – as we mention in the Results (page 6, lines 282) and Discussion (page 8, lines 373-380). It is not possible from the type of data that we have (i.e. field surveillance of resistance under varying environmental conditions) to infer causal relationships. The variable importance measures that we have reported in the Results describe the influence of each variable on the predictions (according to specific definitions of variable importance – page 12 line 584). In the Discussion of our manuscript we draw on the variable importance results to hypothesize about the relationships with the most influential predictor variables (such as ITN coverage) and the reasons why these relationships are spatially variable.

With these points in mind, we agree with the Reviewer that the variables with the highest importance value, such as ITN coverage and rainfall, cover a relatively wide portion of the modelled area, and that this may influence their importance value. It is possible that climatological variables (such as rainfall) delineate regions in space that have common resistance characteristics (perhaps due to unmeasured variables such as genetic similarity). It may not be that rainfall itself is causally related to resistance.

Regarding the Reviewer's suggestion of amalgamating variables representing crops that receive pesticides into a single variable, we believe that this might distort the available data, because we have very little information about how much pesticide each crop type receives or what pesticide type, and whether this differs across different areas. Amalgamation involves removing information, which could be deleterious to predictive performance, and, as explained above, it would not allow us to make conclusive inferences about importance of agricultural land-use in driving resistance trends.

We have added a paragraph to the Discussion (page 8, lines 373-380) to clarify ways in which we can interpret the regional differences in predicted insecticide resistance in relation to the predictor variables included in our model, including variables relating to agricultural pesticide use.

* *The authors have discussed the difference in insecticide resistance between An.gambiae and An.arabiensis. I have 4 points, I wanted to raise on this.*

o *The prediction model gives a good idea of the trends in West Africa as well as predicting factors. The resistance trend in the vectors in West Africa (gamiae/coluzzi) is more homogenous than in the other sibling species in East Africa Arabiensis/gambiae. Could it explain the wider credible interval in the prediction they found in East compare to West Africa?*

This is an interesting point and the reviewer correctly points out that the relative abundance of *An. arabiensis* is higher in east compared to west Africa. We agree that this may lower the prediction accuracy in some parts of the east region, although we don't believe it is the main reason for the wider credible intervals in the east. This is because the credible intervals are widest in Sudan and South Sudan, where the *An. gambiae* complex is made up almost entirely of *An.*

arabiensis. In these areas, we don't have samples with mixed sibling species, and we would expect trends to be more consistent in areas where there is only a single sibling species present. The wider credible intervals in the east will be affected by the relatively low sampling density in this area (see the new Fig. 1 in the main ms).

o The authors include a variable on relative abundance of Arabiensis vs gambiae/coluzzi. which do not seem to contribute to the overall model in East Africa. Please would you be able to explain.

We agree that we would expect resistance trends in an area to be influenced by composition of the sibling species. The variable that we include describing the relative abundance of An. arabiensis vs gambiae/coluzzii is a layer generated from a model that includes predictor variables describing habitat suitability for each sibling species. These variables are climate processes which are also among those included in our insecticide resistance model. Thus we believe that in our analysis, changes in species composition are being captured by the climate variables that we have included as predictors.

o There are reports showing a shift toward An.arabiensis that seems to be associated with IRS and ITN. Would this and lower insecticide resistance prevalence in An.arabiensis explained why ITN coverage is not as strongly predictive for resistance in East Africa compared to West Africa?

We agree that this is certainly possible. In our Discussion (page 7, lines 338-), we stated that "... in several areas of the central and southern parts of east Africa, such as west Tanzania, ITN coverage has been relatively high (>50%) from 2012-2017 [31] but predicted pyrethroid resistance in 2017 is relatively low (Fig. 2D). This may be influenced by the locations where resistance mechanisms first emerged, patterns of subsequent gene flow including restricted flow across the Rift Valley[32, 33, 34], and differences among the sibling species within the *An. gambiae* complex[15, 20]. The distribution of *An. arabiensis* extends further than other species in the complex [23] and this species is known to be more plastic in its feeding behaviour, biting outdoors and feeding on cattle[33]. Thus it is possible that selection for resistance in this species lags behind other members of the complex[35, 36, 37]".

o Contribution of Rainfall variables, please could you explain why do you think there is a difference in importance between West and East Africa. Could rainfall be a proxy for increase in An.gambiae abundance and therefore insecticide resistance? Or?

As discussed in our above responses, we agree that rainfall and other climate variables may act as proxy's for delineating areas of similarity (e.g. with respect to species composition or genetic resistance mechanisms) and we have added the following text to the Discussion:

"We note that the relationships between insecticide resistance and the predictor variables represented in our models do not prove causality. Each variable

interacts with other variables (Figs. S10 & S11) and possibly with variables not included in our analysis. For example, variables describing climate processes were ranked as influential predictors (Fig. 8), but these may delineate areas where resistance trends are similar as a result of an unmeasured process, such as mosquito population structure or species composition"

* *Overall, I would suggest the authors to expand on their discussions especially on the contribution factors and possible explanation of differences between West and East Africa.*

We have expanded our Discussion (lines 368-386) to further interpret the modelled relationships between insecticide resistance and our set of predictor variables. In doing so, we have cautioned against drawing causal conclusions, and we agree with Reviewer 3 that "the careful interpretation of the results is a strength of this paper". Our Discussion now interprets the following relationships:

(i) The influence of ITN coverage on resistance, and its lesser impact in the south-east.
(ii) The higher proportional abundance of *An. arabiensis* in the east and how this may lessen selection for resistance.
(iii) Implications of the relatively low coverage of IRS on the modelled relationships with IRS.
(iv) Interpretation of predicted relationships between resistance and the variables relating to the use and environmental fate of agricultural pesticides.
(v) Modelled relationships between resistance and climate variables.
(vi) The need for additional data on genetic resistance mechanisms to interpret trends in resistance phenotypes.

Minor comments,

* *Why was the variable "Proportional abundance of An. arabiensis to An.coluzzii/gambiae" considered as static? The authors indicated this variable was based on collection from1985 -2015. Would the variable not be able to predict better the resistance if it was included as a yearly or bi-annual variable?*

Annual data on relative abundance are unfortunately not available. Data from 1985 to 2015 were combined and used to define the baseline habitat suitability for each species. This then fed into an analysis of relative abundance prior to the deployment of vector control measures, after deployment of ITNs and after deployment of IRS. The relative abundance data insufficient to track or model this variable through time at every location in west and east Africa.

* *The authors mentioned that insecticide use for agriculture was not available therefore different crops and livestock production were used. How were these 30 crops selected? Is it based on known insecticide use?*

The HarvestChoice project of the International Food Policy Research Institute mapped all major crops grown in SubSaharan Africa. Of these 42 crop maps, 30 showed coverage in our two regions of study and all 30 were included in our model.

* *The authors indicate that "Our predictive maps of mean prevalence of resistance are available to visualise alongside the latest susceptibility test data on the IR mapper website (http://www.irmapper.com), and can guide decisions about resistance management at regional and local levels. In making recommendations, our results will need to be considered in combination with (i) data from resistance monitoring of field samples, including other malaria vector species such as An. funestus; (ii) data on the presence of underlying mechanisms of resistance, and (iii) analyses of the expected impacts of resistance management strategies on malaria prevalence".*

Would the maps and predictors be available for each country? Otherwise I am not convinced that the overall maps and results as presented in the paper would be enough to help decision making at national level.
The IRMapper website allows user to select their country(s) of interest and to export the map(s) of their choice for that country.
The predictor variables are available for all countries from the multiple sources listed in Table S9 of the Supplementary Material.

Reviewer 3:

General comments:
1. Given the exceptional analytic skills and unique data, I wonder if the authors would focus on some additional questions (using regions/time sections that are suitable to address them as applicable) that will "elevate the interest" in their work. For example
(a) Are true "reversals of resistance" evident? How to discern true reversals? If observed, how common are they? What factors may account for them? Are they more common during early evolution phase (when mutations are presumably more costly) and how they can be exploited in management of resistance?
b) are there islands of susceptibility and resistance that are stable. If so where, how long, and what could account for them (see above).
c) is there a way to infer the "lower rate of spatial spread of resistance" (assuming arriving from nearest area) over distance and possibly certain barriers?
d) how unstable are resistance profiles at a location, so if we have several resistance tests in a region that had indicated 51-67% 2,3,4, or up to 7 years ago, how important is to do that test this year if there is no dramatic change in insecticide use and if there was. How such answers change based on availability of a complete time series 10, 50, 100, or 500 km away. So, can we refine the guidance when and where we need new tests.

The questions (a)-(d) ask for a more detailed characterisation of the spread dynamics of resistance, in terms of the stability, the rate of spread and the temporal trajectory of spread (e.g. non-monotonicity and reversals) across different locations. We agree these are very interesting questions that can give

insights into the underlying evolutionary processes. We can use our quantitative predictions of the mean prevalence of resistance, as obtained from our geostatistical models, to address them in part.

We have added a new section to the Results on "Regional and local resistance trends" (page 6, lines 259-272, and Figure 7 and S8-S9) where we present new results that extend our analysis of the temporal trends in resistance to quantify trends at the country level (across 28 countries) and the local level, across the point locations spanning our broader west and east study regions. We have also added a new paragraph to the Discussion (page 8, lines 388-402) where we interpret these new results. The results highlight the regional differences in the predicted dynamics of the phenotype across countries and regions, and show a more diverse range of dynamics in the eastern countries compared to western countries. They also reveal the much more variable, and often non-monotonic, dynamics at the local level. In light of these new results, we provide the following responses to the Reviewer's questions (a)-(d):

a) Predicted regional and local dynamics shows numerous instances of declines, or intermediate plateaus, in the prevalence of resistance phenotypes over time, indicating that non-monotonic temporal trends are possible. The spatial heterogeneity in these trends is understandable as the biological and genetic processes underlying resistance vary geographically, as do environmental selection pressures. We explain in the Discussion (page 8, lines 388-402) that the genetic mechanisms underlying resistance are too complex to allow a mechanistic interpretation of these differing resistance trajectories, and also that shifts in species composition can also affect resistance dynamics. In our original Discussion we identified possible reasons for the broad-scale trends found in our Results, for example the slower spread of resistance in the southern part of east Africa may be a result of geographic barriers to dispersal (e.g. the Rift valley) and regional differences in species composition (e.g. a higher proportion of *An. arabiensis*).
b) Our responses to question a) apply also to question b).
c) We don't believe that our predictive maps can be reliably used to infer the spatial spread of resistance, because whether resistance spreads from a nearby population or arises "de novo" as a novel mechanism cannot be determined from our data. Population genetic studies (e.g. Ag1000g, 2017, *Nature*) indicate cases of rapid and very long-range spatial spread of genetic resistance mechanisms that have a common origin, together with introgression between sibling species within the *An. gambiae* complex. We have added a new paragraph to the Introduction (page 3, lines 105-120) to explain how different resistance mechanisms may have emerged independently, and spread.
d) Our new results (Figures S8 and S9) indicate that resistance trends at point locations can potentially be very variable. The reviewer suggests using spatial extrapolation to predict resistance trends in a given location based on observations from a nearby location. Our geostatistical model does exactly this, and provides a statistical framework that accounts for (i) the influence of spatially varying covariates; (ii) the considerable noisiness of the susceptibility test data, and (iii) the spatiotemporal

autocorrelation structure. Thus we believe that the best predictions of resistance for any spatial location based on nearby data points can be obtained from our maps. We note that temporal forecasting of future resistance trends is a challenging exercise that is beyond the scope of this study. In the Discussion (page 9, lines 400-402) we mention that future testing may be fruitful in areas where resistance is still relatively low, as in these areas spread can still be mitigated.

2. The term "spread of resistance" is complex and may better replaced here or be explained in the outset. Intuitively, it conveys the notion of (1) spread from point A to B, which is not the case here. The authors include (2) the rise in frequency of resistance in the same region, and (3) the spread from unknown multiple sources so that no point A to B can be given. A clarification would be helpful.

We have added the following paragraph to the Introduction (page 3, lines 105-120):

"Pyrethroid resistance has not spread outwards from a single origin and there are several known resistance mechanisms. The mechanisms that have been identified can be classed into target site mutations, upregulation of detoxifying enzymes and cuticular thickening [35, 36, 37, 38]. Each class is associated with multiple genetic variants that confer resistance and there is evidence that at least some of these have arisen independently multiple times across Africa [39]. Once a new variant has arisen, increases in its frequency are driven by selection pressures that differ from place to place, for example, through the spatially varying coverage of pyrethroid use in public health and agriculture [4, 22, 40]. The spread of these genetic variants to new locations is driven by gene flow that also varies with location [30, 41, 42, 43, 44, 45]. Here we consider a species complex and the 'spread' of resistance is further influenced by introgression of resistance genes from one species to another [46, 47, 48, 49, 50, 51]. The geospatial patterns in the pyrethroid resistance phenotype that we are studying here are, therefore, the result of a complex combination of multiple origins of multiple genetic variants that have then spread through gene flow and introgression.".

3. Maps are very good to convey "big picture" of changes in resistance level over time but seem a little too perfect and convey a false notion that all is known. I cannot tell which parts of a map are close to "observed", which show gaps between observed and predicted (how big the gap), and which are "predicted" with no observed to compare with.

We agree about the importance of conveying prediction uncertainty associated with our maps, as well as the spatial coverage of the observed data. Our main Results section emphasises this, as it presents a rigorous statistical quantification of predictive uncertainty, including comprehensive out-of-sample prediction results and maps of prediction credible intervals (Fig. 6) (which were also validated by out-of-sample testing; Fig. S6). In order to further facilitate interpretation of our results with respect to the sampling distribution of the observed data, we have included a new Figure in the main Results (Fig. 1) showing the data point locations and identifying data-rich and data-sparse areas. In the first paragraph Discussion (lines 312-332), we mentioned that the IR

mapper website is providing online visualization tool to allow overlays of the susceptibility test data (for a chosen set of years) on top of our predictive maps of the mean prevalence of resistance.

To address the Reviewer's query about which parts of the map are close-to/far-from the observed values, we plotted the out-of-sample prediction error for each data point on a map (Fig. R1). There is no clear spatial signal in the residuals, and we cannot delineate specific spatial areas where out-of-sample prediction errors are relatively high (or low). Moreover, we note that the interpretation of prediction residuals is complicated by the high noisiness of the susceptibility test data. The prediction credible intervals presented in our Results section (Fig. 6) provide a more reliable assessment of spatial variation in prediction error, because these represent the posterior distributions of the final fitted model (which accounts for data noise).

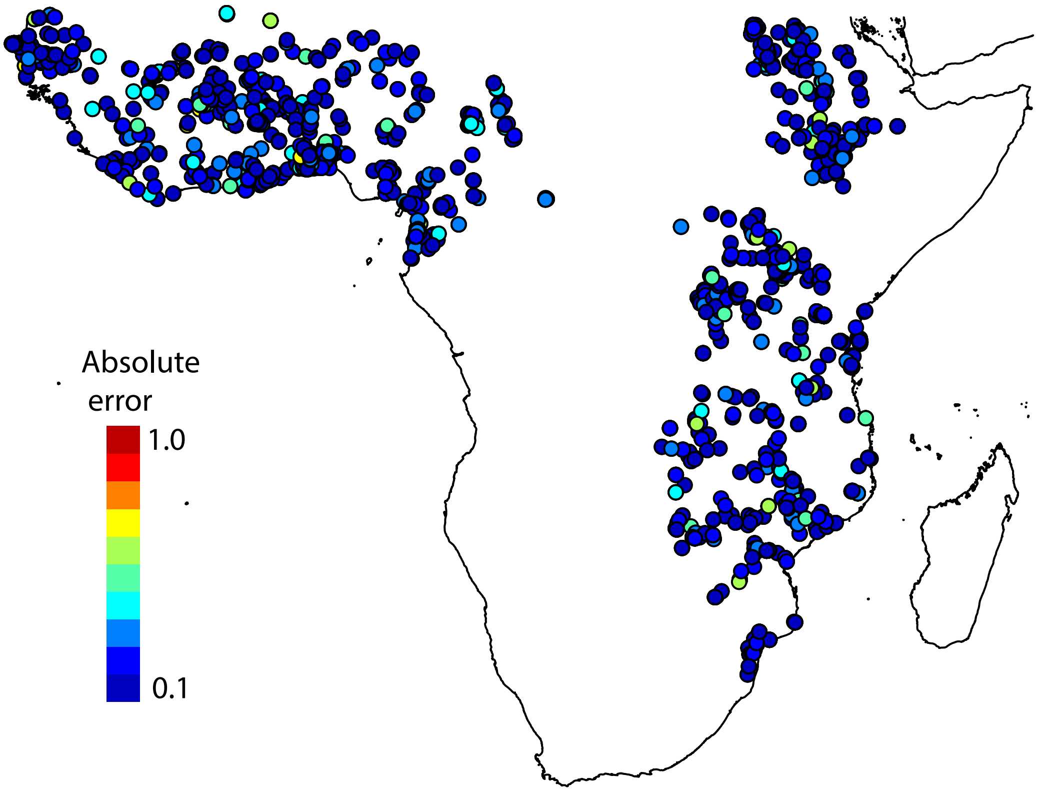

Figure R1. Residual of out-of-sample prediction for each data point.

Specific comments:
Introduction:
- why the range of data used/selected was 2005-17? Given that there is much widespread resistance in 2005, it would be interesting to start earlier, even if it would address smaller areas.

Unfortunately the number of samples in both 2003 and 2004 is extremely sparse (Fig. R2). There is insufficient data to support a robust geospatial analysis of years earlier than 2005, even for smaller areas. The data set that informs our analysis is drawn from a wider data set (published by Moyes et al. 2019, *Scientific Data*) which includes susceptibility test records going back as far as

1955, and interestingly very high resistance levels (low proportional mortality) were recorded even then.

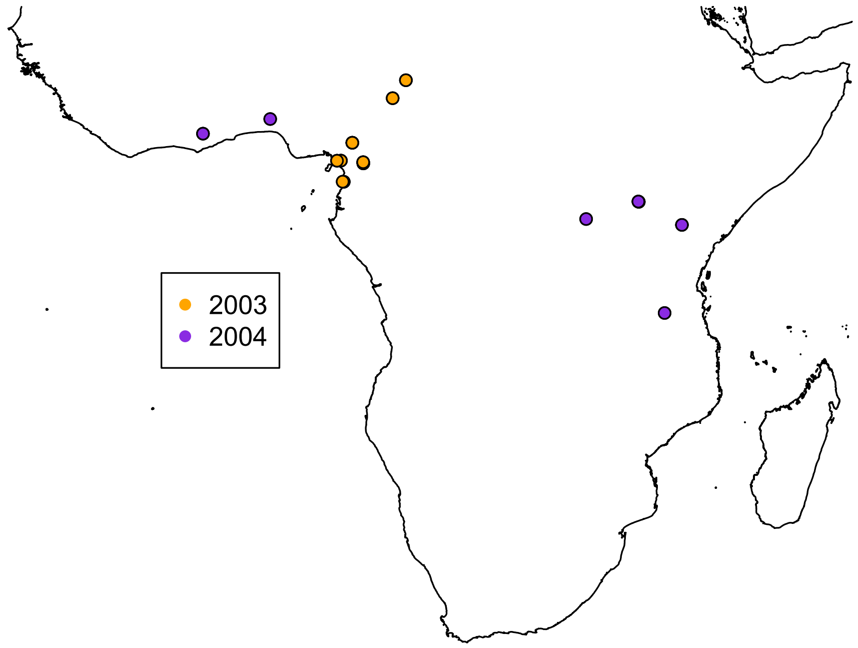

Figure R2. The sampling distribution of susceptibility test results performed in years 2003-2004.

-. *It is mentioned that pyrethroids have been extensively used and given the analysis by different types of pyrethroids, it will help knowing about the difference in sensitivity for each type, mechanism of resistance to each type, and historical use of these types in different areas. Some of this info can form a basis for expectations to judge the maps against.*

There is strong cross-resistance across the different pyrethroid types (deltamethrin, permethrin, lambda-cyhalothrin and alpha-cypermethrin) (Chandre et al. 1999, *J. Am. Mosq. Cont. Assoc.*) and phenotypic resistance levels are typically similar across these pyrethroids (Hancock et al. 2018, *PNAS*). Thus the similarity of our predictive resistance maps across all pyrethroid types is expected. All pyrethroids have the same molecular target site, the voltage-gated sodium channel (*Vgsc*), and point mutations within this target site confer cross-resistance. There are, however, other genetic (metabolic) resistance mechanisms

that are have varying affinities to particular types of pyrethroids (e.g. Ibrahim et al. 2016, *Insect Biochemistry and Molecular Biology*).
We have added the following text to the Introduction (page 3, lines 122-129) about mechanisms of resistance to the different pyrethroids:
"There is evidence that some mechanisms of pyrethroid resistance differ in the level of resistance they confer to different compounds, for example different depletion rates across six pyrethroids compounds have been shown when they were metabolised by eight *Anopheles* cytochrome P450 enzymes under controlled conditions [50]. These differences have not been investigated for all mechanisms of resistance and there is no evidence that the differences seen for individual resistance mechanisms tested in the laboratory translate into diverging patterns of resistance in the field."

We have added the following text to the Introduction (page 2, lines 82-90) to expand on temporal trends in the use of pyrethroids in vector control:

LLIN coverage increased markedly across Africa from 2005 in response the global Roll Back Malaria initiative [22, 23], while IRS coverage has been restricted to much smaller areas [4]. Early insecticide-treated nets used permethrin or deltametrin whereas α-cypermethrin is now the most commonly used pyrethroid in long-lasting insecticide-treated nets. Over the last 20 years, deltamethrin, λ-cyhalothrin and DDT have been used for IRS, with α-cypermethrin first used in mass campaigns in 2003. Since 2015, deltamethrin is the only pyrethroid reported to be used in mass IRS campaigns along DDT and other non-pyrethroid insecticides [4].

- The introduction led me to assume that there were no previous papers on the origin and spread of mechanisms of resistance which were depicted by maps. If this is incorrect, it'd be helpful to refer to their maps and underlying basis.

In response to a similar comment from Reviewer 1 we have added a new paragraph to the Introduction (page 2, lines 67-75) on previous studies of the spatial variation and temporal trends in pyrethroid resistance. We have focused on previous work on the resistance phenotype because that is the focus of our own study although we do now refer to the underlying mechanisms and the origins of these in the new text described above.

Results
I believe if date-stamp is 2005, data were taken only during this year (not from a range, e.g., 2004-2006). Correct?

In our modelling analysis, the date is estimated by the midpoint of the start date and end date of a study, down to the nearest month. The majority of the studies included in our data set were conducted within the same year (>92%). For those studies where collections spanned multiple years, the date will be represented by the mid-point as explained above.

L 198 -214. -
- I assume 'out-of-sample' means removing an observation and using the rest of the

data to predict it. Is 10-fold means that it was repeated 10 times across all observation, one at a time or 90% of the data were used? It needs to be explained.

We have now included an explanation of the 10-fold cross-validation procedure in the Methods section (page 12, lines 572-575) and we refer to this in the Results at page 5, line 223-224, along with a well-known reference to the methodology. We note that *k*-fold cross validation is a necessary part of the Gaussian process stacked generalisation methodology that we have used to create our models (Bhatt et al. 2017, *J. R. S. Interface*), and it is also a widely used approach to assessing prediction error in statistical and machine learning data analyses. We do not use a cross-validation procedure that withholds one data point at a time, firstly because it would be computationally infeasible for our method, and secondly because it would be more affected by the presence of nearby data points than *k*-fold cross-validation (as the reviewer points out).

- I believe that RMSE should be given side by side with MAE, which is more directly applicable to the data and is less susceptible to outliers.

We have now reported the MAE alongside the RMSE in the main text and in Tables S2 and S3. The RMSE is a proper scoring rule, meaning that the score is optimal for the probability distribution that corresponds to the posterior distribution from which the predictions were generated (Gneiting and Raftery, 2007). The MAE is not a proper scoring rule, so we believe that the RMSE is the better score to report in the main text.

- I think this stat is more meaningful conditioned on distance (spatial and temporal if there were multiple observations in the time interval and only 1 was removed) from nearest data points. Further, I'd report the 'adjusted' value to reflect the mean and median distance between observed and predicted that the map shows.

In our responses above we explain that we have used 10-fold cross-validation to assess prediction error, dividing our data into ten (randomly selected) training and test sets, and calculating the out-of-sample prediction error for each data point in each test set. The RMSE is then calculated across all data points in the data set. The RMSE is a well-known scoring rule that we believe will be accessible to readers. We are not aware of any scoring function in the geostatistics literature that conditions, or weights, point-wise prediction errors based on distance from nearby data points, although we appreciate that the model can be expected to have a predictive advantage in areas where there are multiple nearby observations available. However, please see our above responses and Figure R1 where we show that the prediction error doesn't have a strong spatial signal across the data points, and explain that interpretation of prediction error is greatly affected by data noise, as well as the variability of the data and the fitted surfaces.

- I agree that validation of model prediction is vital and fit in the results, although this section is written too technically, and most readers won't be able to interpret it. I suggest keeping the section but transfer parts to the methods and in their

place, provide biological relevant predictions capturing a couple of the better and worse predictions pertaining to relevant space and time points.

We have altered the text of this section (page 5, lines 221-257) to reduce statistical terminology and add intuitive explanations of the statistical concepts. In our responses above we have explained that there isn't a straightforward biological interpretation of prediction error in terms of particular areas and times. Instead we emphasise the importance of the high measurement error associated with standard susceptibility tests to the interpretation of our results on prediction error (page 5-6, lines 230-234). We have revised the text (page 5, lines 246-257) to explain that the maps of the 95% credible intervals of the model predictions (Fig. 4) quantify the model prediction error across space and time, and explain how these can be interpreted together with the new Figure 1 (added following the Reviewer's suggestion above) showing the sampling distribution of the data points.
