# Supplementary material for: Mapping trends in insecticide resistance phenotypes in African malaria vectors
Source: PLoS Biol. 2020 Jun 25;18(6):e3000633. doi: 10.1371/journal.pbio.3000633 (PMC7316233; doi:10.1371/journal.pbio.3000633)
Supplement: Attachment — Submitted filename: Response to reviewers II.pdf [file pbio.3000633.s024.pdf]

Editor:

Many thanks for depositing your data in Figshare; this is much appreciated. However, my understanding is that this largely comprises the "raw" geographical raster data; we also ask that all the numerical values summarized graphically in the figures and results of your paper be made available in one of the following forms:

1) Supplementary files (e.g., excel). Please ensure that all data files are uploaded as 'Supporting Information' and are invariably referred to (in the manuscript, figure legends, and the Description field when uploading your files) using the following format verbatim: S1 Data, S2 Data, etc. Multiple panels of a single or even several figures can be included as multiple sheets in one excel file that is saved using exactly the following convention: S1_Data.xlsx (using an underscore).

2) Deposition in a publicly available repository. Please also provide the accession code or a reviewer link so that we may view your data before publication.

Regardless of the method selected, please ensure that you provide the individual numerical values that underlie the summary data displayed in the following figure panels as they are essential for readers to assess your analysis and to reproduce it: Figs 1A, 3, 5, 7, 8, 9, S4, S5, S6, S8. NOTE: the numerical data provided should include all replicates AND the way in which the plotted mean and errors were derived (it should not present only the mean/average values).

Please also ensure that figure legends in your manuscript include information on where the underlying data can be found (i.e. the Figshare deposition and the bioRxiv DOI), and ensure your supplemental data file/s has a legend.

Please ensure that your Data Statement in the submission system accurately describes where your data can be found.

We have added data files providing the numerical values corresponding to Figures 1A, 3, 5, 7, S4, S5, S6, S8 to our Figshare repository (https://figshare.com/s/00b829f256694ed3c632). The numerical data for Figure 8 is provided in Tables S5 and S6 and the raw data for Figure 9 is available at Moyes et al. [8]. The figure legends have been updated accordingly.

Reviewer #3:

I understand if the authors prefer to expand on that elsewhere, but I would like to hear their comments (2-3 sentences) on the relationships between insecticide resistance and corresponding malaria decline, or lack of decline over that 13-15 years time frame. Their analysis may provide a very valuable if not a decisive answer to this important yet incompletely addressed question.

We agree that this is a critical question. Geospatial analysis of the relationship between trends in insecticide resistance and the burden of malaria is very

complicated as there are numerous factors that influence malaria prevalence at any given place and time, and these vary across space and time. In order to link our results on spatial trends in resistance to what is known about the efficacy of insecticide-based malaria vector control interventions, we have altered last part of the final paragraph of the Discussion as follows:

"Relationships between insecticide resistance and malaria prevalence are currently poorly understood, but there is evidence that resistance can reduce the efficacy of standard pyrethroid-treated LLINs[3], which have played a key role in achieving reductions in malaria prevalence in Africa over 2000-2015[1]. Our maps show marked broad-scale spatial heterogeneity in resistance, motivating the implementation and assessment of a wide range of strategies that target different insecticide resistance and malaria transmission settings, such as next generation LLINs[3,9], as well as rotating insecticide use across different insecticide classes[9]."

Minor points (up to authors to decide if they would like to address):
- L270-2. 'Attenuations and declines in resistance may reflect fitness costs of resistance, or they may also arise due to shifts in the composition of the sibling species that make up the An. gambiae complex (see the Discussion).'
I'd consider adding a the contribution of migration from areas with low resistance.

We have altered this sentence to:
"Attenuations and declines in resistance may reflect fitness costs of resistance, or they may also arise due to shifts in the composition of the sibling species that make up the *An. gambiae* complex, or immigration of mosquitoes from areas where resistance is lower (see the Discussion)."

L 470 'in populations of Gambiae complex mosquito species' , consider revising this fragment. At least, change to ..An. gambiae complex...

We have made this change.
